# Controllable Crystalline Phases of Multi‐Cation Oxides

**DOI:** 10.1002/advs.202412280

**Published:** 2025-04-26

**Authors:** Takafumi Ogawa, Makoto Tanaka, Naoki Kawashima, Taishi Ito, Kei Nakayama, Takeharu Kato, Satoshi Kitaoka

**Affiliations:** ^1^ Nanostructures Research Laboratory Japan Fine Ceramics Center 2‐4‐1 Mutsuno, Atsuta‐ku Nagoya Aichi 456‐8587 Japan; ^2^ Materials Research and Development Laboratory Japan Fine Ceramics Center 2‐4‐1 Mutsuno, Atsuta‐ku Nagoya Aichi 456‐8587 Japan; ^3^ Tokyo University of Technology 1404‐1, Katakura Hachioji Tokyo 192‐0982 Japan

**Keywords:** elemental mapping, first‐principles calculation, high‐entropy oxides, machine learning, rare‐earth titanate, rietveld refinement, scanning transmission electron microscopy

## Abstract

Multi‐cation oxides have been extensively studied over the past decade for various solid‐state applications. The source of their functionality lies in a wide compositional search space derived from countless cation combinations and diverse crystal structures formed in metal oxides. However, due to the vast space and complexity of structure control, material exploration has been limited to dispersed compositions under different synthesis conditions, hindering their systematic understanding and rational design. Here, a crystalline‐phase map of multi‐cation rare‐earth titanates is reported, where three types of crystals, i.e., cubic and hexagonal, and orthorhombic phases, emerge depending on the composition and temperature and exhibit systematic changes. The crystal structures of each phase are thoroughly characterized with X‐ray diffraction, electron microscopy, and first‐principles calculations. The configurational entropies calculated from the crystallographic information support the phase‐boundary shift between hexagonal and orthorhombic phases observed in the phase map. Further, a machine learning procedure is proposed for constructing the map from sparse experimental data, allowing predictive exploration for stable crystalline phases across a large compositional space. These findings may facilitate the design of multi‐cation oxides with a desired structure dispersed in a large search space.

## Graphical abstract

This work demonstrates the control of crystal phases in rare‐earth titanates containing multiple rare‐earth elements. By combining atomic‐level structure characterization techniques, the atomic structures are elucidated, and entropy of mixing for each phase is quantitatively estimated. A crystal‐phase map as a function of temperature and average ionic radius is reproduced and predicted using a machine learning approach.

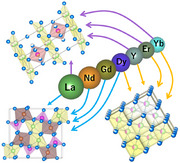

## 1 Introduction

Crystalline oxides accommodating multiple (typically five) cations, frequently called high‐entropy oxides, are among the rapidly growing fields since the work of Rost et al.^[^ ^1^ ^]^ on rock‐salt structure oxides. Recent studies have illustrated the high capability of this approach for various applications;^[^ ^2^, ^3^, ^4^, ^5^, ^6^ ^]^ these include secondary ion batteries,^[^ ^7^, ^8^, ^9^, ^10^ ^]^ optical, electronic and magnetic usages,^[^ ^11^, ^12^, ^13^, ^14^, ^15^, ^16^ ^]^ thermal conductivity reduction,^[^ ^17^, ^18^, ^19^, ^20^, ^21^, ^22^ ^]^ and environmental barrier coatings.^[^ ^23^, ^24^, ^25^ ^]^ While iterative syntheses can aid in the discovery of new functional materials, a vast compositional space remains for exploration of multi‐cation oxides. Additionally, compared with metallic systems, metal oxides can form diverse crystal structures with different local coordination (metal‐oxygen polyhedron) and connectivity. One fundamental issue in the study of multi‐cation oxides has been whether it is possible to synthesize a single crystalline phase rather than mixed phases at a certain composition,^[^ ^1^, ^26^, ^27^, ^28^, ^29^, ^30^, ^31^, ^32^, ^33^ ^]^ as in the cases of multi‐component carbides, nitrides, and borides.^[^ ^34^, ^35^, ^36^, ^37^ ^]^ Furthermore, competition between different crystalline phases has been observed in the multi‐cation oxides: fluorite‐type *A*O_2_ among defect‐fluorite, bixbyite‐type, and pyrochlore‐type structures;^[^ ^31^, ^38^, ^39^ ^]^ perovskite‐type *AB*O_3_ among structures with different symmetry distortions;^[^ ^40^ ^]^ *R*_2_Si_2_O_7_ between the β and γ phases;^[^ ^33^, ^41^ ^]^ and *R*_2_SiO_5_ between the X_1_ and X_2_ phases.^[^ ^42^ ^]^ The dependence of the stable phase on composition of cation‐mixed phases has been often attributed to the configurational entropy, *S*_c_, from the thermodynamic viewpoint.^[^ ^43^, ^44^ ^]^ However, in the previous instances, it remains elusive how the difference in *S*_c_ between the competing phases affects phase stability, because the competing phases have similar cation coordination environments. To achieve a deeper understanding of how *S*_c_ controls phase stability, it is necessary to delve into a systematic behavior involving competition among multiple stable phases with markedly distinct crystal structures and to elaborate methods to estimate *S*_c_ based on crystal structure information.

For the identification of crystalline phases at different compositions and temperatures, it is imperative to prepare samples with a homogeneous distribution under thermal equilibrium. Among various synthesis methods for multi‐component materials, the solid‐state reaction method is a widely used standard,^[^ ^5^ ^]^ although it requires careful selection of powder mixing and grinding procedures, as well as sintering conditions, including treatment temperature and time, to achieve sufficiently mixed samples under thermal equilibrium.^[^ ^45^ ^]^ Alternatively, solution‐based synthesis methods, including sol–gel method, solvothermal technique, nebulized spray pyrolysis, and molecular precursor approach, have gained attention as atom‐up synthetic strategies for preparing well‐mixed powders.^[^ ^5^, ^46^ ^]^ The spray pyrolysis method employed in this work enables precise control of composition with a sufficient mixing of cations and have been already employed in the preparation of powders of few kind of multi‐cation oxides,^[^ ^47^, ^48^, ^49^ ^]^ as well as various rare‐earth oxides.^[^ ^50^, ^51^, ^52^ ^]^

Another fundamental concern related to the role of *S*_c_ is local chemical order, which may reduce entropies, i.e., alter phase stability, and influence materials properties, as investigated for multicomponent alloys.^[^ ^53^, ^54^ ^]^ In complex oxides with two cations, such as defect‐fluorite (*A*_2_ *B*_2_O_7_) and spinel (*AB*_2_O_4_), exhibiting disorder on the cation sublattice from the XRD results, short‐range order within the same sublattice (and the anion sublattice) can exist, as corroborated by neutron total scattering and pair distribution function (PDF).^[^ ^55^, ^56^ ^]^ As for multi‐cation oxides containing four or more cations, homogeneous distribution of cations at the micrometer level has been typically confirmed by the chemical mapping via energy‐dispersive X‐ray spectroscopy (EDS) using scanning transmission electron microscopy (STEM). However, reports on their atomic‐level distribution are relatively scarce. For multi‐cation *A*O compounds with the rock‐salt structure, e.g., Mg_0.2_Ni_0.2_Co_0.2_Cu_0.2_Zn_0.2_O, uniformity of inter‐cationic distance was confirmed by extended X‐ray absorption fine structure (EXAFS),^[^ ^57^ ^]^ though the degree of phase uniformity depends on thermal history and specific composition.^[^ ^1^, ^58^, ^59^, ^60^ ^]^ The combined work of neutron diffraction, PDF, reverse Monte Carlo simulations, and density functional theory (DFT) has revealed the short‐range order on the subnanometer scale in pyrochlore‐type Nd_2_(Ti_0.2_Nb_0.2_Sn_0.2_Hf_0.2_Zr_0.2_)_2_O_7 + *x*_.^[^ ^61^ ^]^ In spinel‐type (Cr, Mg, Fe, Co, Ni)_3_O_4_ with or without including Ga, site selectivity of cations between the tetrahedral and octahedral sites in the crystal has been observed using X‐ray absorption and magnetic circular dichroism, providing the occupancy ratios of cations at each site and their contributions to the configurational entropy.^[^ ^62^ ^]^ In perovskite‐type multi‐cation *AB*O_3_, ordering of cations on the *B* sublattice is suggested from X‐ray diffraction (XRD) and Monte Carlo simulations.^[^ ^29^, ^63^, ^64^ ^]^ Recently, the degree of disordering (ordering) of cations in multi‐cation oxides has been investigated with atomic‐scale observation by STEM‐EDS. For example, atomic‐scale STEM‐EDS visualization of cationic distribution has been reported for perovskite‐type structure^[^ ^65^, ^66^ ^]^ and rock‐salt, pyrochlore, spinel, and perovskite‐type structures.^[^ ^67^ ^]^ However, no clear trend of cation occupancy on the crystal sites has been observed in the atomic‐scale chemical map by STEM‐EDS to the best of our knowledge.

In this study, we focus on the controllability of crystalline phases in multi‐cation oxides and the role of configurational entropy in the crystalline‐phase behavior, via investigation on the crystalline phases in rare‐earth titanates containing multiple *R* elements, (*nR*)_2_TiO_5_, where *R* and *n* denote rare‐earth elements and the number of contained *R* elements. The single *R* series, (1*R*)_2_TiO_5_, forms three polymorphs with distinctly different cation coordination environments at temperatures between 1300 and 1600 °C: orthorhombic, hexagonal, and cubic phases; these are labeled O, H, and C in this paper, respectively. The stable crystalline phase depends on the temperature and ionic radius of the *R* ^3 +^ cations (Figure S1, Supporting Information).^[^ ^68^ ^]^ This series exhibits intriguing properties, such as radiation damage tolerance,^[^ ^69^ ^]^ spin‐ice,^[^ ^70^ ^]^ and low thermal conductivity.^[^ ^50^ ^]^

Here, assuming that the average ionic radius of rare‐earth elements is a good indicator for describing the crystalline phase in the examined oxides, as in the the cases of other multi‐cation oxides,^[^ ^41^, ^71^ ^]^ we systematically prepared samples with equimolar four *R* elements, eq.‐(4*R*)_2_TiO_5_, and their crystalline phases are identified by XRD, exhibiting a variation in crystalline‐phase map that depends on average ionic radius and temperature. For estimating configurational entropy, atomic crystal structure of each single‐phase sample is characterized by using atomic‐scale STEM‐EDS elemental mapping and the Rietveld refinement for synchrotron XRD patterns. During the characterization of the hexagonal phase, a partially renewed crystal model is proposed via analyses of hexagonal Dy_2_TiO_5_ using DFT calculations and Rietveld refinement. The *S*_c_ values calculated based on the obtained crystallographic information are compared and discussed. A machine learning procedure based on the previous and our experimental data of (*nR*)_2_TiO_5_ to reproduce and predict crystalline‐phase maps with a wide range of cation ratios is also presented.

## 2 Results and Discussion

### 2.1 Crystalline‐Phase Map

**Figure 1:**
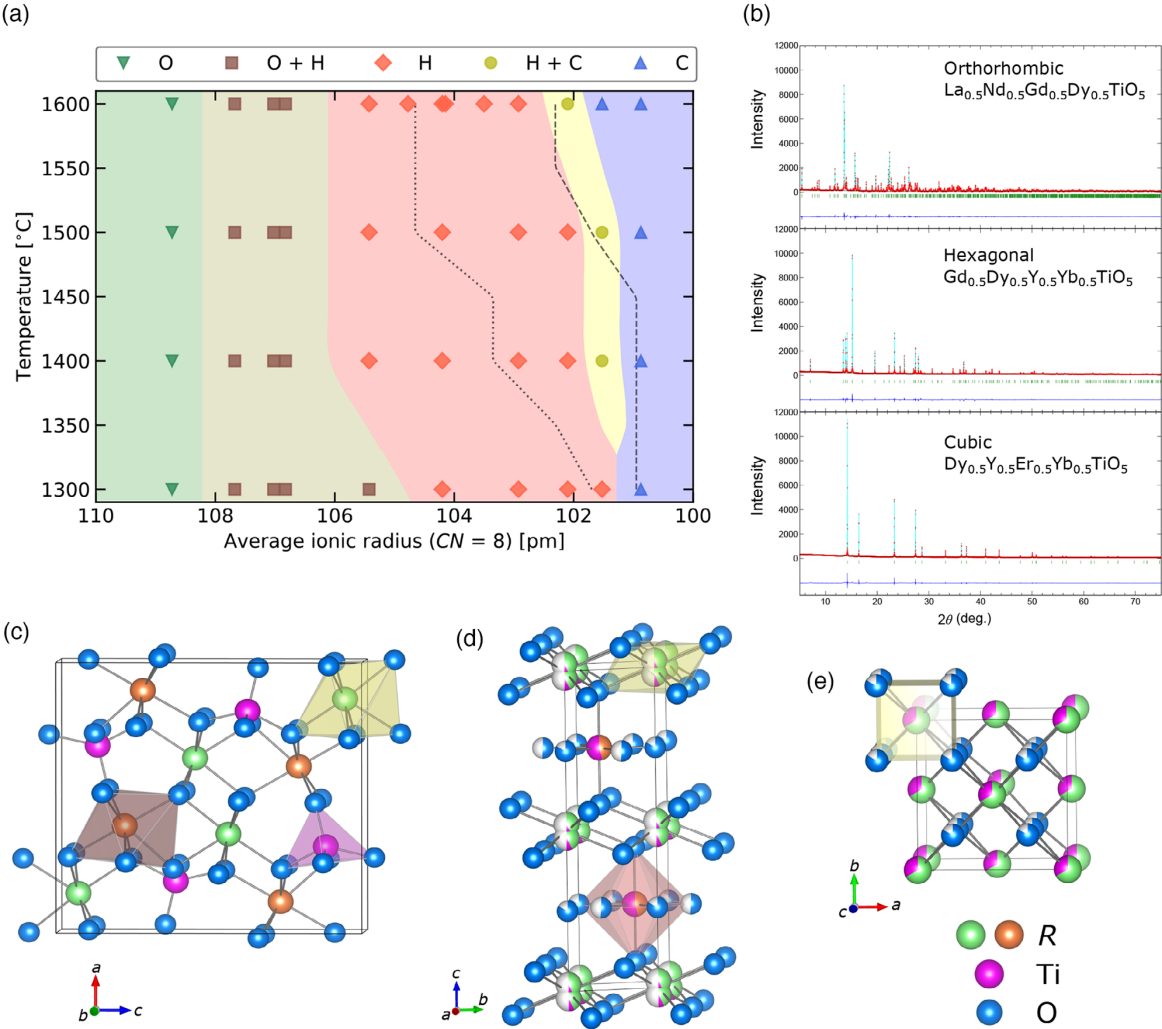
Crystalline phase of rare‐earth titanates containing four equimolar rare‐earth elements, eq.‐(4*R*)_2_TiO_5_. a) Phase map of eq.‐(4*R*)_2_TiO_5_ as a function of temperature and average ionic radius of *R* ^3 +^ with the eight coordination number, where O, H, and C indicate orthorhombic (*Pnma*), hexagonal (*P*6_3_/*mmc*), and cubic ($Fm\bar{3}m$) phases, respectively. The dotted and dashed lines indicate the phase boundaries between the O and H phases and the H and C phases for (1*R*)_2_TiO_5_, respectively. b) Synchrotron XRD patterns of the three types of polymorphs, fitted by the Rietveld method. c,d,e) Crystal structures of the orthorhombic, hexagonal, and cubic phases, respectively. The purple and blue spheres represent titanium and oxygen atoms, respectively. The green and orange spheres represent *R* atoms, where multiple *R* elements on the same lattice site are shown by a single colored sphere.

Ten powder samples of eq.‐(4*R*)_2_TiO_5_ prepared by the spray pyrolysis method are annealed at the examined temperatures for 50 h, while three samples are treated for 2 h at the highest temperature. All examined compositions and identified phases are summarized in **Table** **1**, while the XRD patterns are summarized in Figure S2, Supporting Information. **Figure** **1**a shows the crystalline‐phase map of eq.‐(4*R*)_2_TiO_5_. As the average ionic radius increases, the observed phase changes from the orthorhombic to cubic phases through the hexagonal phase. This sequence is consistent with the single *R*‐element case, but the mixed phases (O + H and H + C) appear in the multi‐cation systems. The occurrence of the mixed phases is not surprising since similar phenomena have been observed in systems involving two *R*‐elements, such as (Gd_1 − *x*_Lu_*x*_)_2_TiO_5_,^[^ ^72^ ^]^ analogous to rock‐salt oxides.^[^ ^58^ ^]^ This behavior can be rationalized from a thermodynamic perspective on mixing.^[^ ^43^ ^]^

**Table 1:** Composition and identified crystal structure of rare‐earth titanates containing four equimolar rare‐earth elements, eq.‐(4*R*)_2_TiO_5_, where O, H, and C indicate orthorhombic, hexagonal, and cubic phases, respectively. Average ionic radius of *R* ^3 +^ with eight coordination numbers, $\langle R_{\mathrm{ion}}^{3+}\rangle$, is expressed in pm.

Composition	$\langle R_{\mathrm{ion}}^{3+}\rangle$	1300 °C	1400 °C	1500 °C	1600 °C
La_0.5_Nd_0.5_Gd_0.5_Dy_0.5_TiO_5_	108.7	O	O	O	O
La_0.5_Nd_0.5_Gd_0.5_Yb_0.5_TiO_5_	107.7	O + H	O + H	O + H	O + H
La_0.5_Nd_0.5_Dy_0.5_Yb_0.5_TiO_5_	107.0	O + H	O + H	O + H	O + H
La_0.5_Nd_0.5_Y_0.5_Yb_0.5_TiO_5_	106.8	O + H	O + H	O + H	O + H
La_0.5_Gd_0.5_Y_0.5_Yb_0.5_TiO_5_	105.4	O + H	H	H	H
La_0.5_Dy_0.5_Y_0.5_Yb_0.5_TiO_5_	104.8	—	—	—	H
Nd_0.5_Gd_0.5_Y_0.5_Yb_0.5_TiO_5_	104.2	—	—	—	H
La_0.5_Y_0.5_Er_0.5_Yb_0.5_TiO_5_	104.2	H	H	H	H
Nd_0.5_Dy_0.5_Y_0.5_Yb_0.5_TiO_5_	103.5	—	—	—	H
Nd_0.5_Y_0.5_Er_0.5_Yb_0.5_TiO_5_	102.9	H	H	H	H
Gd_0.5_Dy_0.5_Y_0.5_Yb_0.5_TiO_5_	102.1	H	H	H	H + C
Gd_0.5_Y_0.5_Er_0.5_Yb_0.5_TiO_5_	101.5	H	H + C	H + C	C
Dy_0.5_Y_0.5_Er_0.5_Yb_0.5_TiO_5_	100.9	C	C	C	C

To investigate the effect of deviation from equimolar mixing, we also synthesized and characterized the structure of La_(2 − *x*)/3_Gd_(2 − *x*)/3_Y_(2 − *x*)/3_Yb_*x*_TiO_5_ (*x* = 0, 0.3, 0.9, 1.5, 1.8), which deviates in Yb content from the composition of La_0.5_Gd_0.5_Y_0.5_Yb_0.5_TiO_5_. The phase map for these compositions is shown in Figure S5, Supporting Information. The figure shows a trend similar to that of eq.‐(4*R*)_2_TiO_5_ (Figure 1a), although a noticeable difference can be observed at around the boundary between orthorhombic and hexagonal phases. In particular, the La_0.567_Gd_0.567_Y_0.567_Yb_0.3_TiO_5_ (*x* = 0.3) sample clearly exhibits single phases with hexagonal and orthorhombic symmetry at 1600 and 1300 °C, respectively. The average ionic radius of the composition corresponds to the coexisting region in the phase map for eq.‐(4*R*)_2_TiO_5_. The results for the samples with non‐equimolar mixing indicate that, while the average ionic radius could represent the crystalline phase in this series, other parameters beyond the average ionic radius are also important for explaining the phase stability over a wide range of compositions. Other descriptors relevant to explaining the phase stability of multi‐cation oxides will be discussed in Section 2.6.

To construct crystal structure models of each phase, we measure synchrotron XRD and perform STEM characterization for three representative samples; La_0.5_Nd_0.5_Gd_0.5_Dy_0.5_TiO_5_, Gd_0.5_Dy_0.5_Y_0.5_Yb_0.5_TiO_5_, and Dy_0.5_Y_0.5_Er_0.5_Yb_0.5_TiO_5_ with orthorhombic, hexagonal, and cubic structures, respectively. While all the samples exhibit homogeneous distribution of cations at the micrometer scale (see Figures S5, S12, and S16, Supporting Information), a partially inhomogeneous distribution of cations at the atomic scale is observed in atomic‐scale STEM‐EDS elemental maps as shown later. The latter information is used to determine cation occupancy ratios in each eq.‐(4*R*)_2_TiO_5_ structure (see Experimental Section for details). Synchrotron XRD patterns and Rietveld refinement results are shown in Figure 1b, indicating a reasonable fit. The applied crystal structure models are shown in Figure 1c–e, where the atomic structures have different coordination environments around the cation sites and different atomic occupancy ratios. In the orthorhombic phase (Figure 1c), the Ti site is coordinated by five O atoms, and two types of similar *R* sites (green and orange) with seven coordinating O atoms are present. In the hexagonal phase (Figure 1d), two cation sites with coexisting Ti and *R* atoms, partially containing green and orange *R* spheres, are coordinated by six and seven O atoms, respectively. The cubic phase (Figure 1e) includes a single type of cation site coordinated by eight O atoms. Such a different coordination environments of cations in each crystal structure can yield different *S*_c_ values among the crystalline phases, as reported for the spinel case.^[^ ^62^ ^]^ Although the dominant role of *S*_c_ in phase stability has been repeatedly asserted in previous studies, the quantitative aspects of the entropy effect remain obscure.^[^ ^44^, ^58^ ^]^

### 2.2 Cubic Phase

The cubic phase of the *R*_2_TiO_5_ series is categorized into defect‐fluorite structure ($Fm\bar{3}m$) based on the XRD patterns, where the cations and anions are fully randomly mixed on each sublattice (Figure 1e). On the other hand, the electron diffraction pattern of the sample displays additional superlattice spots corresponding to pyrochlore‐type ordering (Figure S6, Supporting Information). The pyrochlore structure ($Fd\bar{3}m$) is the well‐known ordered cubic phase derived from the defect‐fluorite structure for *A*_2_ *B*_2_O_7_ compositions and there are a few reports on the macroscopic changes in the degree of cationic ordering between the defect‐fluorite and pyrochlore‐type structures in multi‐cation *A*_2_ *B*_2_O_7_ systems.^[^ ^32^, ^73^ ^]^ Meanwhile, in *A*_2_ *B*O_5_ systems, the formation of long‐range pyrochlore‐type ordering is considered difficult due to the high concentrations of oxygen vacancies and cationic mixing.^[^ ^50^ ^]^ Similar to our observation, nanometer‐order pyrochlore‐type short‐range order within the defect‐fluorite structure has been observed for Ho_2_TiO_5_,^[^ ^74^ ^]^ Yb_2_TiO_5_, and Sm_0.6_Yb_1.4_TiO_5_.^[^ ^75^ ^]^

**Figure 2:**
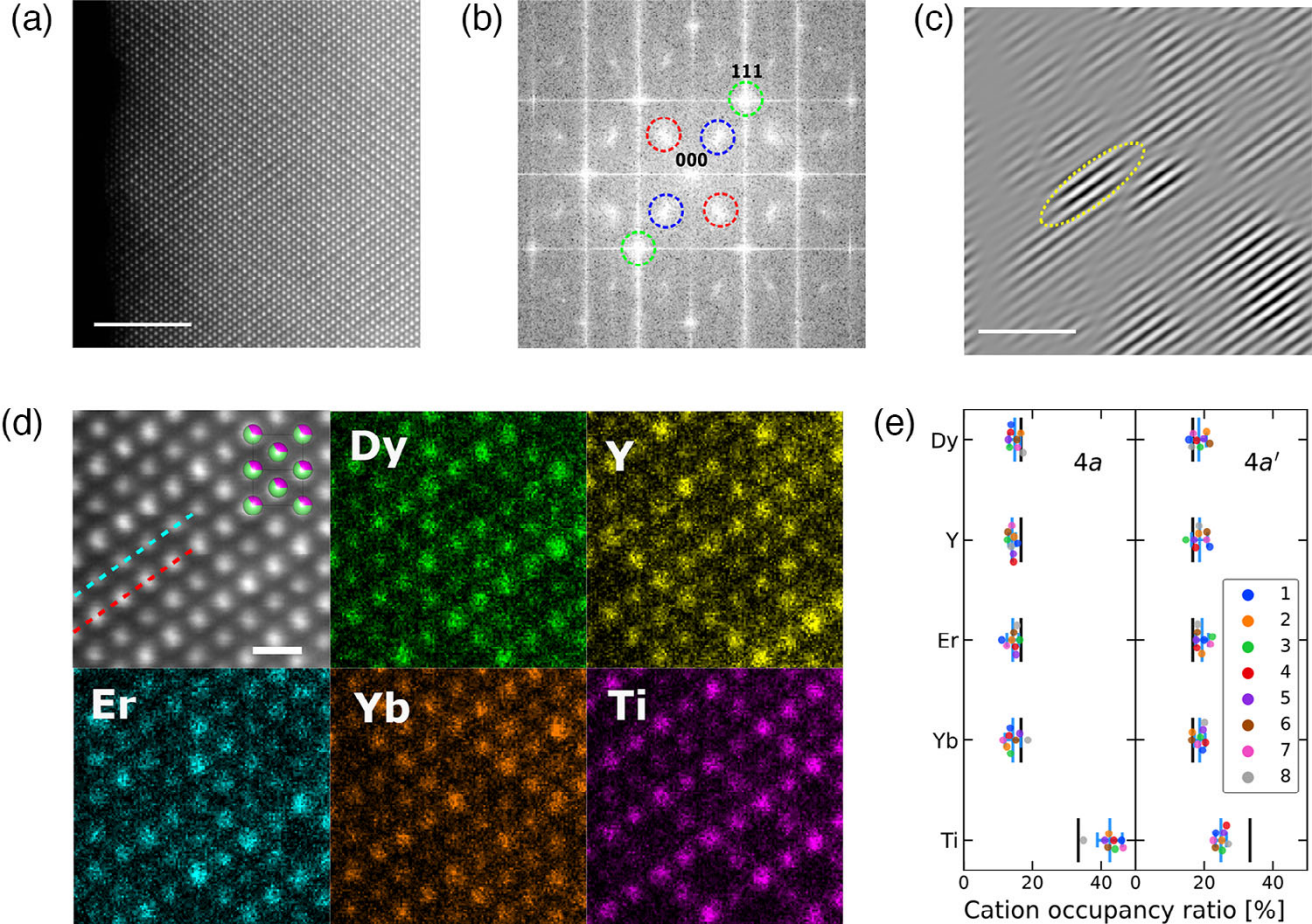
Crystal structure of cubic phase Dy_0.5_Y_0.5_Er_0.5_Yb_0.5_TiO_5_. a) ADF image from the [110] zone axis, where the scale bar indicates 5 nm. b) Fourier‐transformed image of the ADF image (a). The additional spots represented by the red and blue dashed circles between 000 and 111 reflection spots of the defect‐fluorite structure indicate existence of additional short‐range orders of cations. c) Real‐space image by inverse Fourier transformation for the spots indicated by the red circles (b). The yellow dashed ellipse denotes an example of a domain. d) ADF image and EDS elemental maps of each cation, where the scale bar indicates 0.5 nm. In the ADF image, the cations in the cubic structure (Figure 1e) overlap. e) Cation occupancy ratios for the two types of lines, which include Ti‐rich and Ti‐poor columns labeled as 4*a* and 4*a*′, respectively, as indicated by the blue and red dashed lines in the ADF image (d). For each type, eight different lines are measured and plotted (Figure S8, Supporting Information) and the mean values are represented by the blue bars with error bars corresponding to the standard deviations. The black bars indicate the values of the defect‐fluorite model with the nominal composition.

The ordering on the cation sublattice is observed in the annular dark‐field (ADF) image (**Figure** **2**a). The Fourier‐transformed image to the ADF image is shown in Figure 2b, which exhibit the signals of pyrochlore‐type order as similar to the electron diffraction pattern. Inverse Fourier transformation for the pyrochlore spots indicates the arrangement of the pyrochlore‐type domains with sizes up to approximately 10 nm (Figures 2b; Figure S7b, Supporting Information), whereas the inverse transformation for the spots of defect‐fluorite structure shows no modulation (Figure S7a, Supporting Information). Although these results for Dy_0.5_Y_0.5_Er_0.5_Yb_0.5_TiO_5_ are similar to those for Yb_2_TiO_5_ and Sm_0.6_Yb_1.4_TiO_5_,^[^ ^75^ ^]^ it may be concerned that there exists non‐uniform distribution of cations at the atomic scale especially in the multi‐cation case.

In the atomic‐scale EDS maps from the [110] zone‐axis (Figure 2d), compositional variation in the cation columns, i.e., alternating Ti‐rich and Ti‐poor columns in the [111] direction, is directly recognized and corroborates the pyrochlore‐type order due to the partial separation between Ti and other rare‐earth elements (Figure S9, Supporting Information). Figure 2e shows the cation occupancy ratios obtained from the EDS signals along lines with the Ti‐rich (4a) and Ti‐poor(4a′) columns (Figure S8, Supporting Information). As shown in the figure, the Ti ratios (scattered points and blue bars representing average values) deviate from those for the defect‐fluorite structure (black bars). We constructed a pyrochlore‐type structure model using the observed occupancy ratios of each cation (Figure S9, Supporting Information), whereas a defect‐fluorite structure model was constructed based on the macroscopic cation ratios. Rietveld refinement with the pyrochlore‐type structure model results in higher reliability factors than those for the defect‐fluorite structure. Therefore, the sample is considered to approximately form a defect‐fluorite structure, and the refinement parameters for the structure are reported for the cubic phase in Table S2, Supporting Information. Nonetheless, the *S*_c_ values of both models are compared in **Table** **2** to assess the effect of the short‐range order on the entropy, as discussed in Section 2.5.

**Table 2:** Configurational entropy, *S*_c_/*k*_B_, calculated for *R*_2_TiO_5_ with single *R* and equimolar four *R* elements based on the refined crystallographic information.

		*S*_c_/*k*_B_ [atom^−1^]		
Crystal type	Composition	Total	Cation	Oxygen
Cubic‐Fluorite	Yb_2_TiO_5_ ^a)^	0.575	0.240	0.335
Cubic‐Fluorite	Dy_0.5_Y_0.5_Er_0.5_Yb_0.5_TiO_5_	0.917	0.582	0.335
Cubic‐Pyrochlore^b)^	Yb_2_TiO_5_ ^a)^	0.441	0.216	0.225
Cubic‐Pyrochlore^b)^	Dy_0.5_Y_0.5_Er_0.5_Yb_0.5_TiO_5_	0.842	0.546	0.296
Hexagonal	Dy_2_TiO_5_	0.473	0.268	0.205
Hexagonal	Gd_0.5_Dy_0.5_Y_0.5_Yb_0.5_TiO_5_	0.888	0.673	0.215
Orthorhombic	La_2_TiO_5_	0.0	0.0	0.0
Orthorhombic	La_0.5_Nd_0.5_Gd_0.5_Dy_0.5_TiO_5_	0.345	0.345	0.0

a) Calculated based on the previous work.^[^ ^50^ ^]^

b) The reliability factors for the pyrochlore structure are greater than those for the defect‐fluorite structure.

### 2.3 Hexagonal Phase

**Figure 3:**
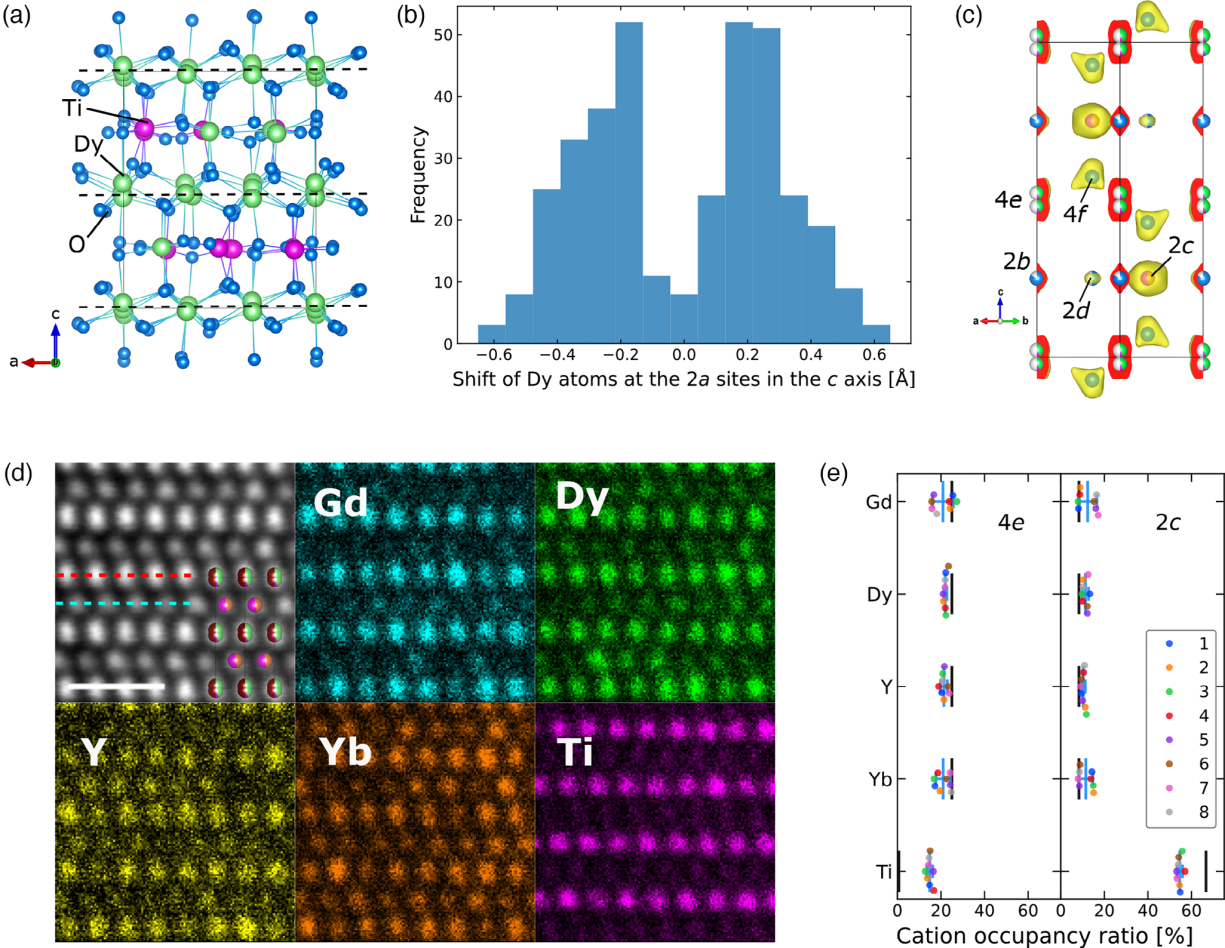
Crystal structure of hexagonal phase Dy_2_TiO_5_ and Gd_0.5_Dy_0.5_Y_0.5_Yb_0.5_TiO_5_. a) Relaxed atomic configuration of stuffed Dy_2_TiO_5_ structure model (B). b) Histogram of the shifts in the positions of the Dy atoms along the *c* axis from the 2*a* site in the previous model, accumulated over the 20 SQS structures of stuffed Dy_2_TiO_5_ structure model (B). c) Electron density map obtained using MEM for the revised hexagonal structure model for the multi‐cation case. d) ADF image and EDS elemental maps of each cation in the multi‐cation oxide, from the [100] zone axes. The scale bar indicates 1 nm. In the ADF image, the cations in the hexagonal structure (Figures 1d) overlap, where the vacant fraction in the cationic spheres is represented in brown for clarity. e) Cation occupancy ratios for *R*‐rich and Ti‐rich lines containing 4*e* and 2*c* sites in the hexagonal structure of the multi‐cation oxide, indicated by the red and blue dashed lines in the ADF image (d), respectively. For each case, eight different lines are measured and plotted (Figure S14, Supporting Information) and the mean values are represented by the blue bars with error bars corresponding to the standard deviations. The black bars in each panel indicate the case in which Ti atoms fully occupy the 2*c* sites with 2/3 occupancy, and the four *R* elements are equally distributed over the remaining portion of each lattice site.

For constructing the hexagonal‐phase structure model of Gd_0.5_Dy_0.5_Y_0.5_Yb_0.5_TiO_5_, we also synthesized the hexagonal‐phase Dy_2_TiO_5_ sample by sintering at 1500°C,^[^ ^76^ ^]^ measured the synchrotron XRD, and characterized it. Although there is a candidate for the hexagonal‐phase structure with *P*6_3_/*mmc* symmetry as applied for Gd_1.8_Lu_0.2_TiO_5_^[^ ^68^ ^]^ and Dy_2_TiO_5_,^[^ ^76^ ^]^ we find inconsistency in density between values from measurements and refinements when using the previous model (Table S3, Supporting Information). To minimize the observed gap, we compared two modified models with DFT calculations (Figure S10 and S11, Supporting Information) and developed a revised structure model (referred as stuffed model (B) in Supporting Information), which has a density consistent with the experimental results (Table S3, Supporting Information). **Figure** **3**a shows an example of the relaxed structure of the revised Dy_2_TiO_5_ model. In these structures, we can observe systematic shifts of Dy atoms in the c‐axis direction at the 2*a* site. Figure figbcb shows the distribution of extracted shift amounts along the *c*‐axis, exhibiting a clear bimodal shape and site‐splitting tendency. To reflect the site splitting, the 2*a* site in the previous model was replaced with the 4*e* site with half occupancy, leading to a significant improvement in the refinement's reliability, as shown in Table S4, Supporting Information.

The structure model of Gd_0.5_Dy_0.5_Y_0.5_Yb_0.5_TiO_5_ shown in Figure 1d is constructed based on the revised model for Dy_2_TiO_5_. The electron diffraction patterns for the hexagonal multi‐cation sample (Figure S13, Supporting Information) have no diffuse scattering, which is different from the previous report for the Sm_1.4_Ti_0.6_TiO_5_:^[^ ^75^ ^]^ Correspondingly, our sample shows no modulation at the micrometer scale. Figure figbcd shows the STEM‐EDS maps of the hexagonal phase at the atomic scale, clearly indicating alternative stacking of the two types of layers including Ti‐rich 2*c* and *R*‐rich 4*e* sites. As shown in the cation occupancy ratios for each layer (Figure 3e), the 4*e* site tends to share nearly equimolar ratios of five cations, and the *R* atoms in the 2*c* site almost equally distribute among the remaining sites after Ti has filled them. The deviations from the black bars in the figure indicate that Ti atoms do not exclusively concentrate on 2*c* site but instead partially occupy the 4*e* site with a large proportion residing on the 2*c* site. Using the above knowledge on the crystal structure model and cationic occupation, the XRD pattern of Gd_0.5_Dy_0.5_Y_0.5_Yb_0.5_TiO_5_ was refined with a high reliability (Table S5, Supporting Information), where its density is consistent with experimental value (Table S3, Supporting Information). The electron density map generated using the maximum entropy method (MEM) emphasizes the importance for improvement in the structure model: Whereas the density map of the previous model exhibits unnatural distributions of oxygen atoms at the 2*b* site (Figure S15, Supporting Information), the density map of the revised structure model shows a more natural distribution (Figure 1c).

### 2.4 Orthorhombic Phase

**Figure 4:**
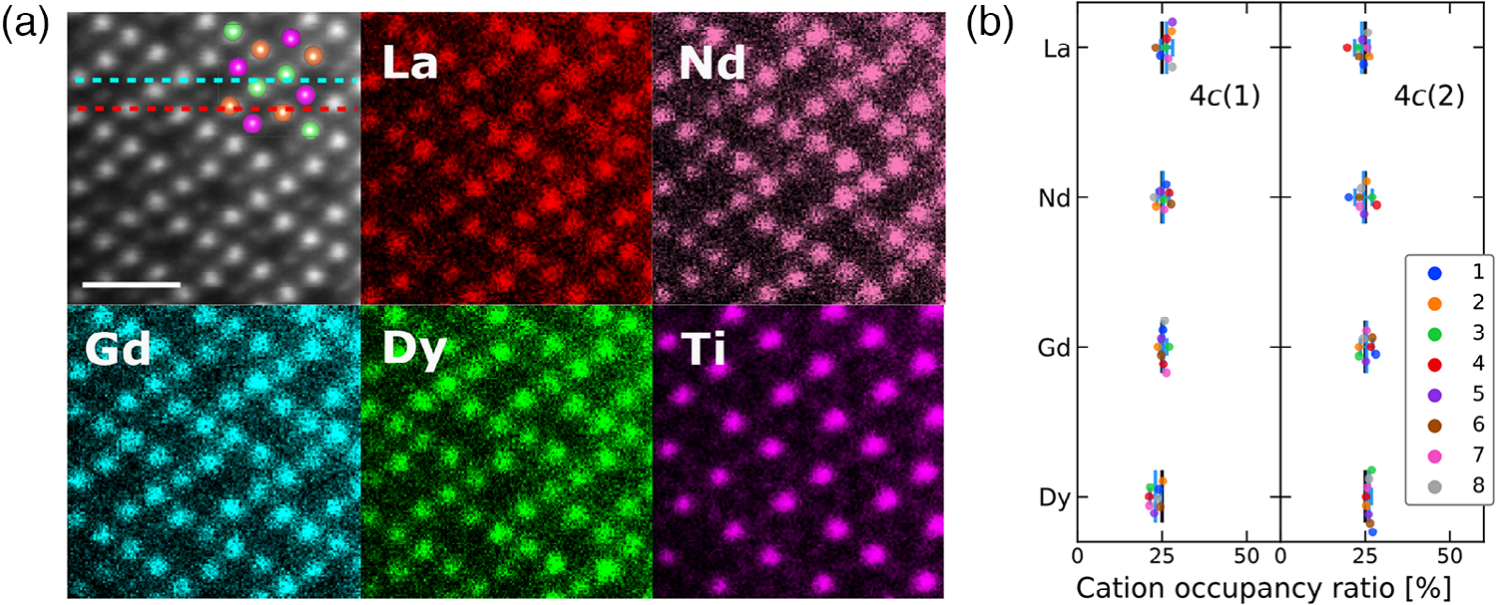
Crystal structure of orthorhombic phase La_0.5_Nd_0.5_Gd_0.5_Dy_0.5_TiO_5_. a) ADF image and EDS elemental maps of each cation, from the [010] zone axes. The scale bar indicates 1 nm. In the ADF image, the cations in the orthorhombic structure (Figures 1c) overlap. b) Cation occupancy ratios for the two types of lines including two different crystallographic *R* sites in the orthorhombic structure, labeled 4*c*(1) and 4*c*(2), respectively, indicated by the blue and red dashed lines in the ADF image (a). Here, the contribution from the Ti sites is omitted due to the clear site separation between Ti and *R* atoms. For each case, eight different lines are measured and plotted (Figure S19, Supporting Information) and the mean values are represented by the blue bars with error bars corresponding to the standard deviations. The black bars indicate the case where the *R* atoms are equally distributed on the two sites.

In the orthorhombic structure, Ti and *R* separately occupy different atomic sites, and two types of *R* sites with similar coordination environments are present (Figure 1c). The structure is confirmed by the electron diffraction pattern (Figure S17, Supporting Information). In the atomic‐scale STEM‐EDS maps of the orthorhombic structure (**Figure** **4**a), the separation between Ti and *R* atoms and homogeneous distribution of *R* elements on the two different *R* sites are clearly observed. For extracting the cation occupancy ratios in the *R* sites, the two lines containing each *R* site are selected and analyzed, where the contributions of Ti columns are omitted. The resulted ratios shown in Figure 4b represent almost equal occupation of the *R* elements. Reflecting this, we constructed an orthorhombic structure model by evenly distributing the *R* elements across the two 4*c* sites. The refinement with this model yielded crystallographic parameters with reasonable reliability (Table S6, Supporting Information).

### 2.5 Configurational Entropy Analysis

By utilizing the site occupancy factors obtained from the refinements, the normalized configurational entropy per atom, *S*_c_/*k*_B_, can be estimated for each crystal structure with four and single *R* compositions (see Experimental Section), in a similar manner as for the spinel structure without oxygen vacancies.^[^ ^77^, ^78^ ^]^ Table 2 represents the obtained *S*_c_/*k*_B_ values. In the cubic case, the entropies for the pyrochlore‐type structure are also listed as a reference, where the refined results exhibit greater reliability factors than those with the defect‐fluorite structure. This preliminary comparison of the two cubic structures indicates a reduction by less than 0.1 in *S*_c_/*k*_B_ due to short‐range order; this reduction amounts are smaller than those over 0.2 in the spinel case where local ordering emerges between cation sites with different coordination environments.^[^ ^77^, ^78^ ^]^ Both *S*_c_/*k*_B_ values for the cubic phases are comparable to the hexagonal values for each number of *R* elements. Based on a comparison of each crystal type, *S*_c_/*k*_B_ of eq.‐(4*R*)_2_TiO_5_ is larger by approximately 0.3–0.4 than those of (1*R*)_2_TiO_5_, which is dominated by the change in the cation contribution. Compared to those of the hexagonal and cubic phases, the *S*_c_/*k*_B_ values of the orthorhombic phase are significantly reduced by approximately 0.45–0.5. It is notable that around half of this difference arises from the contribution of the oxygen sublattice.

In order to examine the relationship between the configurational entropy and phase stability of the multi‐cation oxides, by assuming that the configurational entropy of an (*nR*)_2_TiO_5_ phase is determined by the ratios of rare‐earth elements and crystalline phase and independent of the detailed composition, entropy of mixing (Δ*S*_mix_) for eq.‐(4*R*)_2_TiO_5_ is calculated using the following equation:

(1) $$\Delta S_{\mathrm{mix}}=S_{c}\left(\mathrm{eq}.-{\left(4R\right)}_{2}{\mathrm{TiO}}_{5};\alpha \right)-\frac{1}{4}\sum_{R}S_{c}\left({\left(1R\right)}_{2}{\mathrm{TiO}}_{5};\beta_{\mathrm{MS}}\right).$$

Here, *S*_c_(Comp.; α) indicates the configurational entropy of oxides with the composition (Comp.) in the α phase, as listed in Table 2, and β_MS_ indicate the most stable phase in the composition. Since the change in the free energy (Δ*G*_mix_) of mixing in a solid‐solution crystal is given by Δ*G*_mix_ = Δ*H*_mix_ − *T*Δ*S*_mix_, where Δ*H*_mix_ denotes the enthalpy of mixing,^[^ ^43^ ^]^ a lower Δ*H*_mix_ and higher Δ*S*_mix_ contribute to the enhancement of crystalline‐phase stability. The calculated entropies of mixing in **Table** **3** show that Δ*S*_mix_/*k*_B_ of the orthorhombic phase are relatively lower than those of the hexagonal and cubic phases for each composition, while the values for the hexagonal and cubic phases are similar. Notably, the orthorhombic phase exhibits a destabilizing tendency by the cation mixing for Dy_0.5_Y_0.5_Er_0.5_Yb_0.5_TiO_5_ and Gd_0.5_Dy_0.5_Y_0.5_Yb_0.5_TiO_5_. These reductions in Δ*S*_mix_/*k*_B_ for the orthorhombic phase are likely responsible for the large change in the boundary between orthorhombic and hexagonal phases on the phase map (Figure 1a). In the present case, the origin of the behavior seems to lie not only in differences in compositional complexity, but also in differences in crystallographic complexity.

**Table 3:** Entropy of mixing, Δ*S*_mix_/*k*_B_, calculated for the three crystalline phases of eq.‐(4*R*)_2_TiO_5_ based on the configurational entropy of each phase (Table 2), where bold values correspond to the stable phases of each composition.

	*ΔS*_mix_/*k*_B_ [atom^−1^]		
Composition	Ortho.	Hexa.	Cubic(F)
Dy_0.5_Y_0.5_Er_0.5_Yb_0.5_TiO_5_	−0.179	0.364	**0.393**
Gd_0.5_Dy_0.5_Y_0.5_Yb_0.5_TiO_5_	−0.033	**0.508**	0.537
La_0.5_Nd_0.5_Gd_0.5_Dy_0.5_TiO_5_	**0.227**	0.770	0.799

It may be worth discussing the validity of the magnitudes of the entropy of mixing and their differences between different phases by comparing them with the transition entropy between two polymorphs. Although transition entropy data for the phase transitions of *R*_2_TiO_5_ are not available, the order of entropy change in the transition between orthorhombic and hexagonal phases could be estimated based on free energy data and the transition temperature for Sm_2_TiO_5_.^[^ ^79^ ^]^ When we assume no enthalpy change during the transition, the transition entropy is estimated to be 0.22*k*_B_. This value is of the same order of magnitude as our results in Tables 2 and 3, although the enthalpy should be taken into account in reality. For further discussion, the structural phase transitions of other oxides are considered for comparison. Available transition entropies per atom for rare‐earth sesquioxides (*R*_2_O_3_) and other few oxides lie in the range between −0.1*k*_B_ and 0.6*k*_B_,^[^ ^43^, ^80^ ^]^ and the above estimates for *R*_2_TiO_5_ are on the same order. These quantitative comparisons suggest that the entropy differences arising from the configurational entropies for the multi‐cation and also unmixed oxides have appreciable effects on structural phase transitions. However, the entropies are not a single factor determining crystalline‐phase stability, and contributions from the enthalpy of mixing and other energy factors, such as vibrational enthalpy and entropy, also need to be quantitatively evaluated for a more comprehensive and accurate understanding.^[^ ^44^ ^]^ Additionally, the procedure for estimating the entropy terms above becomes difficult when applied to multi‐cation oxides with aliovalent or multivalent cations, as their incorporation can cause a deviation in oxygen content from the nominal stoichiometry value. One known approach to prevent this deviation is to select the composition (cation ratios) to maintain the stoichiometric oxygen content.^[^ ^29^ ^]^

### 2.6 Machine Learning Classification

**Figure 5:**
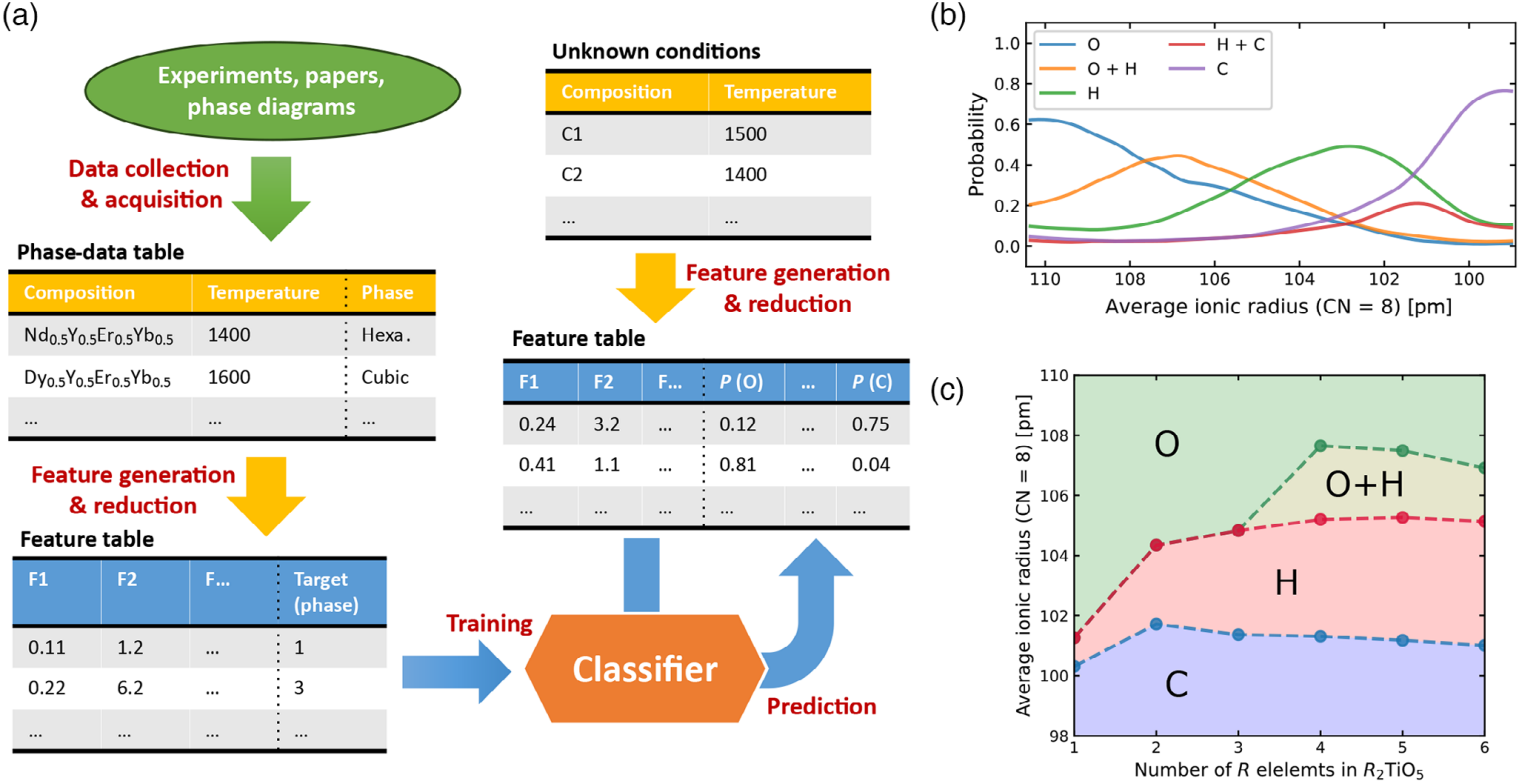
Machine learning classification of the crystalline phases of (*nR*)_2_TiO_5_. a) Overall flow diagram for the classification of the crystalline phases. The composition of *R* elements and temperature are chosen as primitive descriptors, and the temperature is in range from 1300 to 1600 °C with 50 °C separation. The phase data table is translated to the feature table, which is fed to the classifier. The trained classifier outputs the probability of each single and mixed phase, i.e., O, O + H, H, H + C, and C, for any given feature input. b) Probability distribution averaged over all compositions of eq.‐(4*R*)_2_TiO_5_ as a function of the average ionic radius, at *T* = 1300 °C. Here, the phase with the highest probability was adopted as the stable phase at each average radius, to construct the phase map. c) Dependence of the crystalline phases on the number of *R* elements (*n*) for (*nR*)_2_TiO_5_ with equimolar *R* elements, at *T* = 1300 °C.

As observed in the eq.‐(4*R*)_2_TiO_5_ case, the phase stability of multi‐cation oxides is expected to vary continuously in a highly complex compositional space. However, experimental exploration has been limited to a small part of the total due to finite resources. To extend the phase map experimentally obtained for *R*_2_TiO_5_, we constructed a machine‐learning classification procedure, shown schematically in **Figure** **5**a. At the first step in the classification scheme, available experimental data including our data are collected and extracted to construct the phase‐data table that includes composition, temperature, and crystalline phase, as summarized in Figure S20 and Table S7, Supporting Information. Then, the descriptors (composition and temperature) are transformed, for each data, into numerous features based on elemental descriptors, e.g., ionic radius and standard enthalpy of formation of *R*_2_O_3_ (Supporting Note S1, Supporting Information). This procedure to generate descriptors is analogous to the general‐purpose approach,^[^ ^81^ ^]^ where a wide variety of chemical attributes are used to generate descriptors without prior selection. In contrast, a number of studies applying machine learning to high‐entropy ceramics^[^ ^33^, ^35^, ^37^, ^71^, ^82^, ^83^, ^84^ ^]^ and high‐entropy metals^[^ ^85^ ^]^ have employed several attributes or a few descriptors, such as the deviation in ionic radius of cations, to explain phase stability and other physical properties. Although learning with a small number of descriptors selected through feature‐importance evaluation was also examined in our application, better performance and explainable model could not be achieved. The feature table is fed into the classifier in the training step. Before adopting the scheme for prediction, a tree‐base classifier was selected via the assessment of various classifiers using cross‐validation (Figure S23, Supporting Information).

In the prediction step, the table of compositions and temperatures to be predicted is transformed into the feature table in the same manner as in the training step. The predictions of the trained classifier for the feature table provide the probabilities of each crystalline phase (see Experimental Section for more details). The distribution of the probabilities is averaged at each temperature for each phase to determine phase boundaries (Figure S24, Supporting Information). Figure 5b shows an example of probability distribution at *T* = 1300 °C. By this procedure, the eq.‐(4*R*)_2_TiO_5_ phase map is reproduced as shown in Figure S25a, Supporting Information. When our data are omitted from the training data, the classification near the O–H phase boundary changes significantly as shown in Figure S25b, Supporting Information, confirming that our data with *n* = 4 are essential for improving the prediction accuracy of orthorhombic and hexagonal phases. The predicted *n*‐dependence of the stable phases for a series of (*nR*)_2_TiO_5_ with equimolar *R* elements shows a tendency for the hexagonal phase area to expand with increasing *n* (Figure 5c). This may reflect the larger entropy of mixing for the hexagonal phase compared to that for the orthorhombic phase, as already discussed. Since the accuracy of predictions in this procedure depends on the quantity, dispersion, and qualities of data, developments of automated synthesis and characterization^[^ ^42^, ^86^, ^87^ ^]^ are expected to enhance the efficacy of machine‐learning‐based exploration of high‐dimensional phase maps for multi‐cation oxides.

## 3 Conclusion

The experimental investigation of the crystalline phases of *R*_2_TiO_5_ has demonstrated that the multi‐cation approach leads to continuous rather than abrupt changes in the crystalline‐phase stability, possibly driven by the difference in entropy of mixing between competing crystalline phases. The atomic‐level characterization of cation distribution within the crystal lattice, combined with STEM‐EDS elemental mapping and refinements of diffraction patterns, have aided in determining occupancy ratios and elucidating the role of configurational entropy. In the case examined here, the competing crystal structures have highly different occupation environments for cations and oxygen atoms. The differences in site preference are considered the origin of the variation of the entropy values and, consequently, phase stability, while the pyrochlore‐type local ordering in the cubic phase may not have a significant effect. Based on the observed continuity, unexplored regions can be predicted using a classifier trained on sparse experimental data points. The produced phase map for a multi‐cation oxide series is expected to accelerate the discovery of new materials with attractive properties. By integrating high‐throughput or automated generation of experimental data under thermal equilibrium, the developed approaches will provide an optimized pathway for exploring functional multi‐cation oxides.

## 4 Experimental Section

Raw powders of (4*R*)_2_TiO_5_ were synthesized using ultrasonic spray pyrolysis, which allows fine control of the composition, as shown elsewhere.^[^ ^47^, ^50^ ^]^ Aqueous precursor solutions were obtained by mixing *R*(NO_3_)_3_ · *m*H_2_O (*m* = 4–6) and aqueous solutions of TiCl_4_. The raw powders were calcined at 1400 °C for 2 h in air. The powders were subsequently annealed at 1300–1600 °C for 50 h to investigate the crystalline phase at each temperature. To obtain sintered bodies, the calcined powders were sintered by hot pressing at 50 MPa in Ar at 1200–1300 °C for 30 min and then annealed in air at 1400–1600 °C for 4–9 h. Dense sintered bodies were obtained for cubic Dy_0.5_Y_0.5_Er_0.5_Yb_0.5_TiO_5_, hexagonal Gd_0.5_Dy_0.5_Y_0.5_Yb_0.5_TiO_5_, and Dy_2_TiO_5_, and their densities were 6.80, 6.80, and 7.26 g/cm^3^, respectively. The cation molar ratios of all the samples were measured via inductively coupled plasma optical emission spectroscopy (ICP‐OES) and reflected in the following structural characterization.

To identify the crystalline phase of the powders annealed for 50 h at each temperature, XRD data were collected with a RINT2500 diffractometer (Rigaku) with Cu‐Kα radiation. The crystal structures of the eq.‐(4*R*)_2_TiO_5_ powders made by grinding the samples were accurately analyzed via powder XRD patterns obtained via a synchrotron X‐ray detector (PILATUS 100K, Dectris Ltd.) with an energy of 16.9 keV, a step size of 0.01, and an exposure time of 300 s at BL5S2 of the Aichi SR. Rietveld refinements were performed by the RIETAN‐FP program.^[^ ^88^ ^]^ The electron density distributions were obtained via MEM analysis from observed structural factors resulting from Rietveld refinements. The limited‐memory Broyden–Fletcher–Goldfarb–Shanno (L‐BFGS) algorithm^[^ ^89^ ^]^ implemented in Dysnomia^[^ ^90^ ^]^ was used to obtain the exact solutions. MEM analyses and whole‐pattern fitting were alternately repeated to minimize biases in favor of the structural models for the Rietveld analyses. The crystal structures and electron density distributions were drawn by VESTA.^[^ ^91^ ^]^

The configurational entropy per atom, *S*_c_, of a crystal structure was estimated by using the following equation:

(2) $$S_{c}=-\frac{k_{B}}{N_{s}^{0}}\sum_{\sigma }^{N_{s}}g_{\sigma }\sum_{i}^{N_{e,\sigma }}f_{\sigma ,i}\ln f_{\sigma ,i},$$

where *f*_σ, *i*_, *g*_σ_, σ, and *i* denote site occupancy factor, the multiplicity, a crystal site, and an element, respectively; here, *i* explicitly includes vacancies to satisfy $\sum_{i}^{N_{e,\sigma }}f_{\sigma ,i}=1$. *N*_s_ and *N*_e, σ_ are the number of site types in the crystal and the number of elements on site σ, respectively. $N_{s}^{0}$ is the total number of sites, calculated using $N_{s}^{0}=\sum_{\sigma }^{N_{s}}g_{\sigma }$.

For the characterization by STEM, the sintered materials were ground into fine powders using an agate mortar and pestle. The powders were placed on a cupper grid with carbon film. STEM imaging and EDS analysis of the powders were performed on an atomic‐resolution scanning transmission electron microscope with an aberration corrector (JEM‐ARM300F2 GRAND ARMTM2, JEOL Ltd., Tokyo) equipped with large‐sized twin silicon drift detector system (158 mm^2^), operated at 200 kV. The probe‐forming aperture and collected semi‐angles for ADF imaging were 24.5 and 54–220 mrad, respectively, and the electron probe current was set to 120 pA. The acquisition of EDS elemental maps including drift correction and EDS analysis, was performed by using Analysis Station ver. 4.41.0.10 (JEOL Ltd., Tokyo).

The EDS analysis provides a quantitative estimation of the cation ratios of the multi‐cation oxides at low magnification, although the values of the cation ratios at the atomic scale may include certain measurement deviations. To minimize the measurement variations, the cation ratios measured from the ICP‐OES analysis were used to scale the cation ratios of each line area in the EDS analysis with scaling factors between the ratios from the EDS signals of the whole observed range and those from ICP‐OES data. In the case of the orthorhombic phase La_0.5_Nd_0.5_Dy_0.5_Gd_0.5_TiO_5_, since the EDS peaks of Ti (K_α_ = 4.51 keV) and La (L_α_1 = 4.65 keV) were close to each other, clearly separated elemental maps of these atoms were difficult to obtain by using the same procedure described above. Therefore, the elemental maps of Ti and La were generated by using energy ranges between 4.39 and 4.56 keV and between 4.62 and 4.79 keV, respectively, with DigitalMicrograph software (GATAN, Inc., US) (Figure S18, Supporting Information).

DFT calculations of Dy_2_TiO_5_ models were performed using the VASP code.^[^ ^92^, ^93^ ^]^ For the exchange‐correlation functional, the GGA‐PBE functional was adopted.^[^ ^94^ ^]^ Based on the previous Dy_2_TiO_5_ model (Figure S10a, Supporting Information),^[^ ^68^ ^]^ atoms on fractional occupancy sites were distributed within the 2× 2 ×1 supercell via the procedure of special‐quasirandom structure (SQS) procedure.^[^ ^95^ ^]^ The energy cutoff and ***k***‐mesh grids were set to 520 eV and 2× 2 ×2, respectively. The lattice constants and internal positions were optimized until the forces on the atoms were greater than 0.01 eV/Å. The relaxed energies and densities (volumes) were averaged over 20 different SQS structures for each model.

In the classification scheme for the crystalline phases in *R*_2_TiO_5_, the training dataset consisted of collected and extracted data from the database and literature (Figure S20 and Table S7, Supporting Information), as well as our data (Table 1 and Table S1, Supporting Information). For feature generation, elemental descriptors were used (Supporting Note S1, Supporting Information). To select the classifier model, we compared available algorithms using the scikit‐learn package in python,^[^ ^96^ ^]^ where the input dataset was normalized with the “Standard Scaler” module of scikit‐learn before being fed into classification. The hyperparameters of each classifier were optimized using the optuna library^[^ ^97^ ^]^ and cross‐validation with different separations was used. Several classifiers achieved 90% or better accuracy (Figure S23, Supporting Information). Among those, the extra‐tree classifier was selected to produce the phase maps. The probability distribution of each phase, which was output from the classifier, was averaged over the average ionic radius at each temperature (Figure S24, Supporting Information).

## Conflict of Interest

The authors declare no conflict of interest.

## Supporting information

Supporting Information

## Data Availability

The data that support the findings of this study are available from the corresponding author upon reasonable request.

## References

[1] C. M. Rost , E. Sachet , T. Borman , A. Mobballegh , E. C. Dickey , D. Hou , J. L. Jones , S. Curtarolo , J.‐P. Maria , Nat. Commun. 2015, 6, 8485. 26415623 10.1038/ncomms9485 PMC4598836

[2] C. Oses , C. Toher , S. Curtarolo , Nat. Rev. Mater. 2020, 5, 295.

[3] H. Xiang , Y. Xing , F. Dai , H. Wang , L. Su , L. Miao , G. Zhang , Y. Wang , X. Qi , L. Yao , H. Wang , B. Zhao , J. Li , Y. Zhou , J. Adv. Ceram. 2021, 10, 385.

[4] Y. Ma , Y. Ma , Q. Wang , S. Schweidler , M. Botros , T. Fu , H. Hahn , T. Brezesinski , B. Breitung , Ene. Environ. Sci. 2021, 14, 2883.

[5] G. N. Kotsonis , S. S. I. Almishal , F. M. dos Santos Vieira , V. H. Crespi , I. Dabo , C. M. Rost , J.‐P. Maria , J. Am. Ceram. Soc. 2023, 106, 5587.

[6] V. Álvarez‐Montaño , M. Kumar , S. Sharma , R. K. Chourasia , P. Kumar , J. Siqueiros , O. R. Herrera , Mater. Lett. 2023, 349, 134785.

[7] Z. Lun , B. Ouyang , D.‐H. Kwon , Y. Ha , E. E. Foley , T.‐Y. Huang , Z. Cai , H. Kim , M. Balasubramanian , Y. Sun , J. Huang , Y. Tian , H. Kim , B. D. McCloskey , W. Yang , R. J. Clément , H. Ji , G. Ceder , Nat. Mater. 2021, 20, 214. 33046857 10.1038/s41563-020-00816-0

[8] R. Zhang , C. Wang , P. Zou , R. Lin , L. Ma , L. Yin , T. Li , W. Xu , H. Jia , Q. Li , S. Sainio , K. Kisslinger , S. E. Trask , S. N. Ehrlich , Y. Yang , A. M. Kiss , M. Ge , B. J. Polzin , S. J. Lee , W. Xu , Y. Ren , H. L. Xin , Nature 2022, 610, 67. 36131017 10.1038/s41586-022-05115-z

[9] Y. Zeng , B. Ouyang , others, G. Ceder , Science 2022, 378, 1320. 36548421 10.1126/science.abq1346

[10] F. Ding , C. Zhao , D. Xiao , X. Rong , H. Wang , Y. Li , Y. Yang , Y. Lu , Y.‐S. Hu , J. Am. Chem. Soc. 2022, 133, 8286. 10.1021/jacs.2c02353 35472274

[11] B. Zhao , Z. Yan , Y. Du , L. Rao , G. Chen , Y. Wu , L. Yang , J. Zhang , L. Wu , D. W. Zhang , R. Che , Adv. Mater. 2020, 35, 2210243. 10.1002/adma.202210243 36606342

[12] Z. J. Corey , P. Lu , G. Zhang , Y. Sharma , B. X. Rutherford , S. Dhole , P. Roy , Z. Wang , Y. Wu , H. Wang , A. Chen , Q. Jia , Adv. Sci. 2022, 9, 2202671. 10.1002/advs.202202671 PMC9561869 36026570

[13] A. R. Mazza , E. Skoropata , Y. Sharma , J. Lapano , T. W. Heitmann , B. L. Musico , V. Keppens , Z. Gai , J. W. Freeland , T. R. Charlton , M. Brahlek , A. Moreo , E. Dagotto , T. Z. Ward , Adv. Sci. 2022, 9, 2200391. 10.1002/advs.202200391 PMC8981892 35150081

[14] G. Zhang , Y. Wu , Int. J. Appl. Ceram. Technol. 2022, 19, 644.

[15] L. Xia , Z. Mao , X. Wang , J. Zhu , J. Xie , Z. Wang , W. Hu , J. Mater. Chem. C 2023, 11, 9899.

[16] B. Yang , Y. Liu , S. Lan , L. Dou , C.‐W. Nan , Y.‐H. Lin , J. Appl. Phys. 2023, 133, 110904.

[17] J. L. Braun , C. M. Rost , M. Lim , A. Gin , D. H. Olson , G. N. Kotsonis , G. Stan , D. W. Brenner , J.‐P. Maria , P. E. Hopkins , Adv. Mater. 2018, 30, 1805004. 10.1002/adma.201805004 PMC9486463 30368943

[18] A. J. Wright , Q. Wang , S.‐T. Ko , K. M. Chung , R. Chen , J. Luo , Scr. Mater. 2020, 181, 76.

[19] M. Ridley , J. Gaskins , P. Hopkins , E. Opila , Acta Mater. 2020, 195, 698.

[20] X. Liu , P. Zhang , M. Huang , Y. Han , N. Xu , Y. Li , Z. Zhang , W. Pan , C. Wan , J. Eur. Ceram. Soc. 2023, 43, 6407.

[21] Y. Zhang , J. Zhu , H. Zou , K. Yang , M. Li , H. Wang , J. He , J. Alloys Compnds. 2024, 976, 172942.

[22] H. Vakilifard , H. Shahbazi , A. C. Liberati , R. B. N. Saraswathy , R. S. Lima , M. D. Pugh , C. Moreau , J. Thermal Spray Technol. 2024, 33, 447.

[23] Y. Dong , K. Ren , Y. Lu , Q. Wang , J. Liu , Y. Wang , J. Eur. Ceram. Soc. 2019, 39, 2574.

[24] K. Bryce , Y.‐T. Shih , L. Huang , J. Lian , J. Eur. Ceram. Soc. 2023, 43, 6461.

[25] R. B. Nair , D. Brabazon , npj Mater. Deg. 2024, 8, 44. 10.1038/s41529-024-00462-w PMC11045441 38682040

[26] S. Jiang , T. Hu , J. Gild , N. Zhou , J. Nie , M. Qin , T. Harrington , K. Vecchio , J. Luo , Scr. Mater. 2018, 142, 116.

[27] A. Sarkar , R. Djenadic , D. Wang , C. Hein , R. Kautenburger , O. Clemensand , H. Hahn , J. Eur. Ceram. Soc. 2018, 38, 2318.

[28] K. C. Pitike , S. KC , M. Eisenbach , C. A. Bridges , V. R. Cooper , Chem. Mater. 2020, 32, 7507.

[29] L. Tang , Z. Li , K. Chen , C. Li , X. Zhang , L. An , J. Am. Ceram. Soc. 2021, 104, 1953.

[30] J. He , G. He , J. Liu , J. Tao , J. Eur. Ceram. Soc. 2021, 41, 6080.

[31] L. Spiridigliozzi , C. Ferone , R. Cioffi , G. Dell'Agli , Acta Mater. 2021, 202, 181.

[32] H. Yang , G. Lin , H. Bu , H. Liu , L. Yang , W. Wang , X. Lin , C. Fu , Y. Wang , C. Zeng , Ceram. Int. 2022, 48, 6956.

[33] Y. Fan , Y. Bai , Q. Li , Z. Lu , D. Chen , Y. Liu , W. Li , B. Liu , npj Comput. Mater. 2024, 10, 95.

[34] K. Balasubramanian , S. V. Khare , D. Gall , Acta Mater. 2018, 152, 175.

[35] D. G. Sangiovanni , K. Kaufmann , K. Vecchio , Sci. Adv. 2023, 9, eadi2960. 37703369 10.1126/sciadv.adi2960 PMC10499311

[36] D. R. Lowry , J. R. Boro , M. Blea‐Kirby , N. R. Valdez , S. R. Bishop , J. Am. Ceram. Soc. 2023, 106, 7078.

[37] S. Divilov , H. Eckert , D. Hicks , C. Oses , C. Toher , R. Friedrich , M. Esters , M. J. Mehl , A. C. Zettel , Y. Lederer , E. Zurek , J.‐P. Maria , D. W. Brenner , X. Campilongo , S. Filipović , W. G. Fahrenholtz , C. J. Ryan , C. M. DeSalle , R. J. Crealese , D. E. Wolfe , A. Calzolari , S. Curtarolo , Nature 2024, 625, 66. 38172364 10.1038/s41586-023-06786-y PMC10764291

[38] L. Spiridigliozzi , C. Ferone , R. Cioffi , G. Dell'Agli , Ceram. Int. 2023, 49, 7662.

[39] L. Spiridigliozzi , C. Ferone , R. Cioffi , G. Dell'Agli , Materials 2023, 2023, 2219.

[40] J. Zhang , S. Liu , Z. Tian , Y. Zhang , Z. Shi , Materials 2023, 2023, 2214.

[41] Y. Luo , L. Sun , J. Wang , T. Du , C. Zhou , J. Zhang , J. Wang , Nat. Commun. 2023, 14, 1275. 36882392 10.1038/s41467-023-36947-6 PMC9992687

[42] H. Meng , P. Wei , Z. Tang , H. Yu , Y. Chu , J. Materiomics 2024, 10, 738.

[43] S. J. McCormack , A. Navrotsky , Acta Mater. 2021, 202, 1.

[44] S. S. Aamlid , M. Oudah , J. Rottler , A. M. Hallas , J. Am. Chem. Soc. 2023, 145, 5991. 36881986 10.1021/jacs.2c11608

[45] A. D. Dupuy , M. R. Chellali , H. Hahn , J. M. Schoenung , J. Eur. Ceram. Soc. 2021, 41, 4850.

[46] M. A. Buckingham , J. M. Skelton , D. J. Lewis , Cryst. Growth Des. 2023, 23, 6998. 10.1021/acs.cgd.3c00712 PMC10557048 37808901

[47] A. Sarkar , R. Djenadic , N. J. Usharani , K. P. Sanghvi , A. S. G. Venkata SK Chakravadhanula , H. Hahn , S. S. Bhattacharya , J. Eur. Ceram. Soc. 2017, 37, 747.

[48] A. Sarkar , L. Velasco , D. Wang , Q. Wang , G. Talasila , L. de Biasi , C. Kübel , T. Brezesinski , S. S. Bhattacharya , H. Hahn , B. Breitung , Nat. Commun. 2018, 9, 3400. 30143625 10.1038/s41467-018-05774-5 PMC6109100

[49] M. R. Chellali , A. Sarkar , S. H. Nandam , S. S. Bhattacharya , B. Breitung , H. Hahn , L. Velasco , Scr. Mater. 2019, 166, 58.

[50] K. Asai , M. Tanaka , T. Ogawa , U. Matsumoto , N. Kawashima , S. Kitaoka , F. Izumi , M. Yoshida , O. Sakurada , J. Solid State Chem. 2020, 287, 121328.

[51] M. Tanaka , T. Matsudaira , E. Kawai , N. Kawashima , U. Matsumoto , T. Ogawa , M. Takeuchi , S. Kitaoka , J. Ceram. Soc. Jpn. 2021, 129, 22.

[52] S. Kitaoka , M. Tanaka , N. Kawashima , T. Ito , D. Yokoe , T. Kato , T. Ogawa , N. Yamazaki , N. Hosoya , T. Nakamura , J. Am. Ceram. Soc. 2023, 106, 4863.

[53] X. Chen , Q. Wang , Z. Cheng , M. Zhu , H. Zhou , P. Jiang , L. Zhou , Q. Xue , F. Yuan , J. Zhu , X. Wu , E. Ma , Nature 2021, 592, 712. 33911276 10.1038/s41586-021-03428-z

[54] S. Chen , Z. H. Aitken , S. Pattamatta , Z. Wu , Z. G. Yu , D. J. Srolovitz, P. K. Liaw , Y.‐W. Zhang , Nat. Commun. 2021, 2, 4953. 10.1038/s41467-021-25264-5 PMC8368001 34400654

[55] J. Shamblin , M. Feygenson , J. Neuefeind , C. L. Tracy , F. Zhang , S. Finkeldei , D. Bosbach , H. Zhou , R. C. Ewing , M. Lang , Nat. Mater. 2016, 15, 507. 26928636 10.1038/nmat4581

[56] E. C. O'Quinn , K. E. Sickafus , R. C. Ewing , G. Baldinozzi , J. C. Neuefeind , M. G. Tucker , A. F. Fuentes , D. Drey , M. K. Lang , Sci. Adv. 2020, 6, eabc2758. 32923649 10.1126/sciadv.abc2758 PMC7455179

[57] C. M. Rost , Z. Rak , D. W. Brenner , J.‐P. Maria , J. Am. Ceram. Soc. 2017, 100, 2732.

[58] M. Fracchia , et al., Nat. Commun. 2022, 13, 2977. 35624095 10.1038/s41467-022-30674-0 PMC9142508

[59] S. S. I. Almishal , L. Miao , Y. Tan , G. N. Kotsonis , J. T. Sivak , N. Alem , L.‐Q. Chen , V. H. Crespi , I. Dabo , C. M. Rost , S. B. Sinnott , J.‐P. Maria , J. Am. Ceram. Soc. 2024, 108, e20223.

[60] S. S. I. Almishal , J. T. Sivak , G. N. Kotsonis , Y. Tan , M. Furst , D. Srikanth, V. H. Crespi , V. Gopalan , J. T. Heron , L.‐Q. Chen , C. M. Rost , S. B. Sinnott , J.‐P. Maria , Acta Mater. 2024, 279, 120289.

[61] B. Jiang , C. A. Bridges , R. R. Unocic , K. C. Pitike , V. R. Cooper , Y. Zhang , D.‐Y. Lin , K. Page , J. Am. Chem. Soc. 2021, 143, 4193. 33352040 10.1021/jacs.0c10739

[62] G. H. J. Johnstone , M. U. González‐Rivas , K. M. Taddei , R. Sutarto , G. A. Sawatzky , R. J. Green , M. Oudah , A. M. Hallas , J. Am. Chem. Soc. 2022, 144, 20590. 36321637 10.1021/jacs.2c06768

[63] J. Ma , K. Chen , C. Li , X. Zhang , L. An , Ceram. Int. 2021, 47, 24348.

[64] A. V. Motseyko , N. V. Ter‐Oganessian , J. Alloys Compnds. 2024, 976, 172945.

[65] L. Su , et al., Nat. Commun. 2022, 13, 2358. 35487934 10.1038/s41467-022-30018-y PMC9055071

[66] A. Sarkar , D. Wang , M. V. Kante , L. Eiselt , V. Trouillet , G. Iankevich , Z. Zhao , S. S. Bhattacharya , H. Hahn , R. Kruk , Adv. Mater. 2023, 35, 2207436. 10.1002/adma.202207436 36383029

[67] H. Gao , N. Guo , Y. Gong , L. Bai , D. Wang , Q. Zheng , Nanoscale 2023, 15, 19469. 37987086 10.1039/d3nr05176e

[68] Y. F. Shepelev , M. A. Petrova , Inorg. Mater. (Engl. Transl.) 2008, 44, 1496.

[69] R. D. Aughterson , G. R. Lumpkin , K. L. Smith , J. M. Cairn , J. Am. Ceram. Soc. 2020, 103, 5536.

[70] G. C. Lau , R. S. Freitas , B. G. Ueland , B. D. Muegge , E. L. Duncan , P. Schiffer , R. J. Cava , Nat. Phys. 2006, 2, 249.

[71] Q. Lan , J. Ma , S. Li , K. Chen , C. Li , L. An , Ceram. Int. 2023, 49, 21091.

[72] M. A. Petrova , A. S. Novikova , D. P. Romanov , R. G. Grebenshchikov , Izv. Akad. Nauk SSSR, Neorg. Mater; 1986, 22, 1225.

[73] A. J. Wright , Q. Wang , C. Hu , Y.‐T. Yeh , R. Chen , J. Luo , Acta Mater. 2021, 211, 116858.

[74] G. Lau , T. McQueen , Q. Huang , H. Zandbergen , R. Cava , J. Solid State Chem. 2008, 181, 45.

[75] R. D. Aughterson , G. R. Lumpkin , M. de los Reyes , N. Sharma , C. D. Ling , B. Gault , K. L. Smith , M. Avdeev , J. M. Cairney , J. Solid State Chem. 2014, 213, 182.

[76] R. D. Aughterson , N. J. Zaluzec , G. R. Lumpkin , Acta Mater. 2021, 204, 116518.

[77] T. Parida , A. Karati , K. Guruvidyathri , B. S. Murty , G. Markandeyulu , Scr. Mater. 2020, 178, 513.

[78] A. Sarkar , B. Eggert , R. Witte , J. Lill , L. Velasco , Q. Wang , J. Sonar , K. Ollefs , S. S. Bhattacharya , R. A. Brand , H. Wende , F. M. F. de Goot , O. Clemens , H. Hahn , R. Kruk , Acta Mater. 2022, 226, 117581.

[79] W. Gong , Y. Liu , Z. Luo , J. Alloys Compnds. 2021, 860, 158429.

[80] M. Zinkevich , Prog. Mater. Sci. 2007, 52, 597.

[81] L. Ward , A. Agrawal , A. Choudhary , C. Wolverton , npj Comput. Mater. 2016, 2, 16028.

[82] J. Zhang , X. Xiang , B. Xu , S. Huang , Y. Xiong , S. Ma , H. Fu , Y. Ma , H. Chen , Z. Wu , S. Zhao , Current Opinion in Solid State Mater. Sci. 2023, 27, 101057.

[83] K. C. Pitike , A. Macias , M. Eisenbach , C. A. Bridges , V. R. Cooper , Chem. Mater. 2022, 34, 1459.

[84] J. Liu , A. Wang , P. Gao , R. Bai , J. Liu , B. Du , C. Fang , J. Am. Ceram. Soc. 2024, 107, 1361.

[85] W. Chen , A. Hilhorst , G. Bokas , S. Gorsse , P. J. Jacques , G. Hautier , Nat. Commun. 2023, 14, 2856. 37208345 10.1038/s41467-023-38423-7 PMC10199023

[86] Q. Wang , L. Velasco , B. Breitung , V. Presser , Adv. Ene. Mater. 2021, 11, 2102355.

[87] J. Chen , S. R. Cross , L. J. Miara , J.‐J. Cho , Y. Wang , W. Sun , Nat. Synth. 2024, 3, 606.

[88] F. Izumi , K. Momma , Solid State Phenom. 2007, 130, 15.

[89] J. Nocedal , Math. Comput. 1980, 35, 773.

[90] K. Momma , T. Ikeda , A. A. Belik , F. Izumi , Powder Diffr. 2013, 28, 184.

[91] K. Momma , F. Izumi , J. Appl. Crystallogr. 2011, 44, 1272.

[92] G. Kresse , J. Furthmüller , Computat. Mater. Sci. 1996, 6, 15.

[93] G. Kresse , D. Joubert , Phys. Rev. B 1999, 59, 1758.

[94] J. P. Perdew , A. Ruzsinszky , G. I. Csonka , O. A. Vydrov , G. E. Scuseria , L. A. Constantin , X. Zhou , K. Burke , Phys. Rev. Lett. 2008, 100, 136406. 18517979 10.1103/PhysRevLett.100.136406

[95] A. van de Walle , P. Tiwary , M. de Jong , D. L. Olmsted , M. Asta , A. Dick , D. Shin , Y. Wang , L.‐Q. Chen , Z.‐K. Liu , Calphad 2013, 42, 13.

[96] F. Pedregosa , G. Varoquaux , A. Gramfort , V. Michel , B. Thirion , O. Grisel , M. Blondel , P. Prettenhofer , R. Weiss , V. Dubourg , J. Vanderplas , A. Passos , D. Cournapeau , M. Brucher , M. Perrot , É. Duchesnay , J. Machine Learn. Res. 2011, 12, 2825.

[97] T. Akiba , S. Sano , T. Yanase , T. Ohta , M. Koyama , In Proceedings of the 25th ACM SIGKDD International Conference on Knowledge Discovery and Data Mining 2019.

