# Supplementary material for: Controllable Crystalline Phases of Multi‐Cation Oxides
Source: Adv Sci (Weinh). 2025 Apr 26;12(20):2412280. doi: 10.1002/advs.202412280 (PMC12120718; doi:10.1002/advs.202412280)
Supplement: Supplementary file 1 — Supporting Information [file ADVS-12-2412280-s001.pdf]

ADVANCED SCIENCE
Open Access

Supporting Information

for *Adv. Sci.*, DOI 10.1002/advs.202412280

Controllable Crystalline Phases of Multi-Cation Oxides

Takafumi Ogawa, Makoto Tanaka*, Naoki Kawashima, Taishi Ito, Kei Nakayama, Takeharu Kato and Satoshi Kitaoka**

Supplorting Information:
Controllable Crystalline Phases of Multi-Cation Oxides

Takafumi Ogawa,[1,*] Makoto Tanaka,[2,†] Naoki Kawashima,[2] Taishi Ito,[1] Kei Nakayama,[1] Takeharu Kato,[1] and Satoshi Kitaoka[2,3,‡]

[1]*Nanostructures Research Laboratory, Japan Fine Ceramics Center, 2-4-1 Mutsuno, Atsuta-ku, Nagoya, 456-8587, Aichi, Japan*

[2]*Materials Research and Development Laboratory, Japan Fine Ceramics Center, 2-4-1 Mutsuno, Atsuta-ku, Nagoya, 456-8587, Aichi, Japan*

[3]*Tokyo University of Technology, 1404-1, Katakura, Hachioji, Tokyo, 192-0982, Japan*

[*] t_ogawa@jfcc.or.jp

[†] m_tanaka@jfcc.or.jp

[‡] kitaoka@jfcc.or.jp

Crystalline-phase map of $(1R)_2TiO_5$

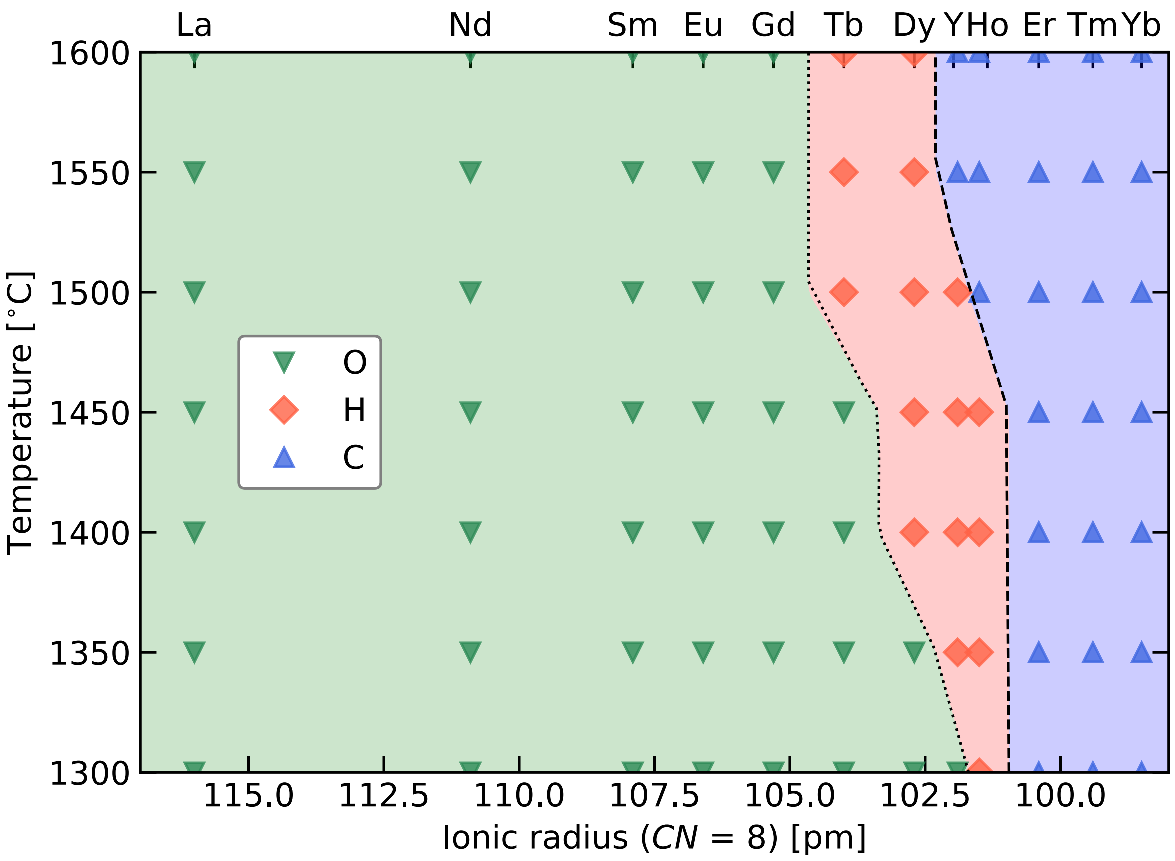

Figure S1. Collected crystalline-phase map of R_2TiO_5 with a single R element as a function of the ionic radius with a coordination number of eight. The dotted and dashed lines indicate the phase boundaries between the O and H phases and the H and C phases, respectively, which are overlaid on other phase maps as a reference. Data sources are listed in Table S6 and categorized as db-1.

Crystalline phases of equimolar $(4R)_2\mathrm{TiO}_5$

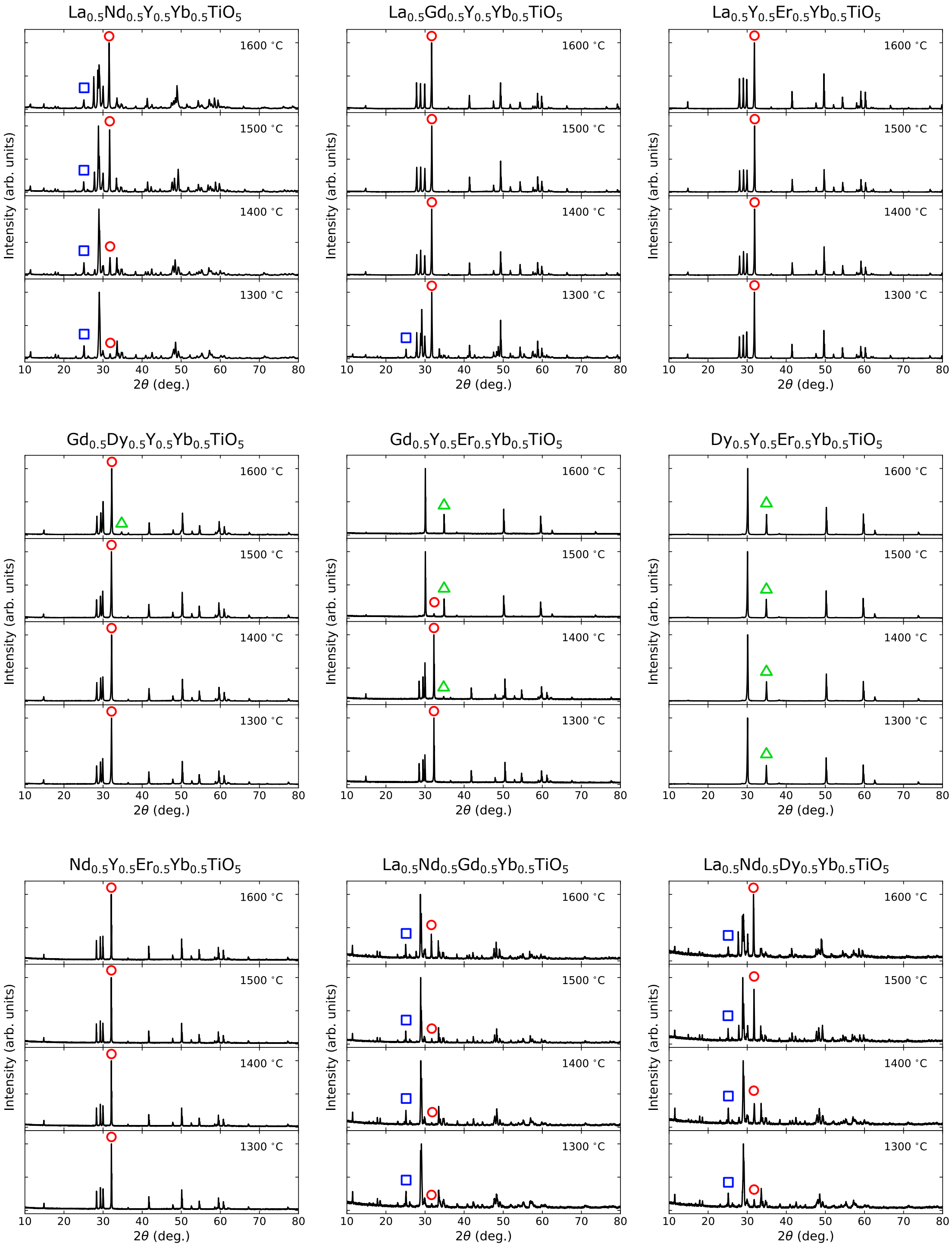

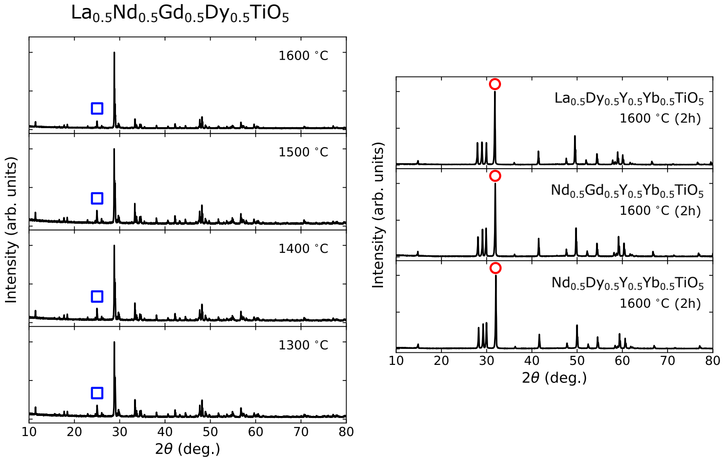

Figure S2. X-ray diffraction (XRD) pattern of $(4R)_2TiO_5$ with equimolar four R elements, prepared via thermal treatment for 50 h at each temperature, unless otherwise specified for the treated time. The green triangles and red circles, and blue squares represent peaks denoting the presence of cubic, hexagonal, and orthorhombic phases, respectively. The identified phases are listed in Table 1 and plotted in the phase map in Figure 1a of the main text.

Crystalline phases of $La_{(2-x)/3}Gd_{(2-x)/3}Y_{(2-x)/3}Yb_xTiO_5$ (x = 0, 0.3, 0.5, 0.9, 1.5, 1.8)

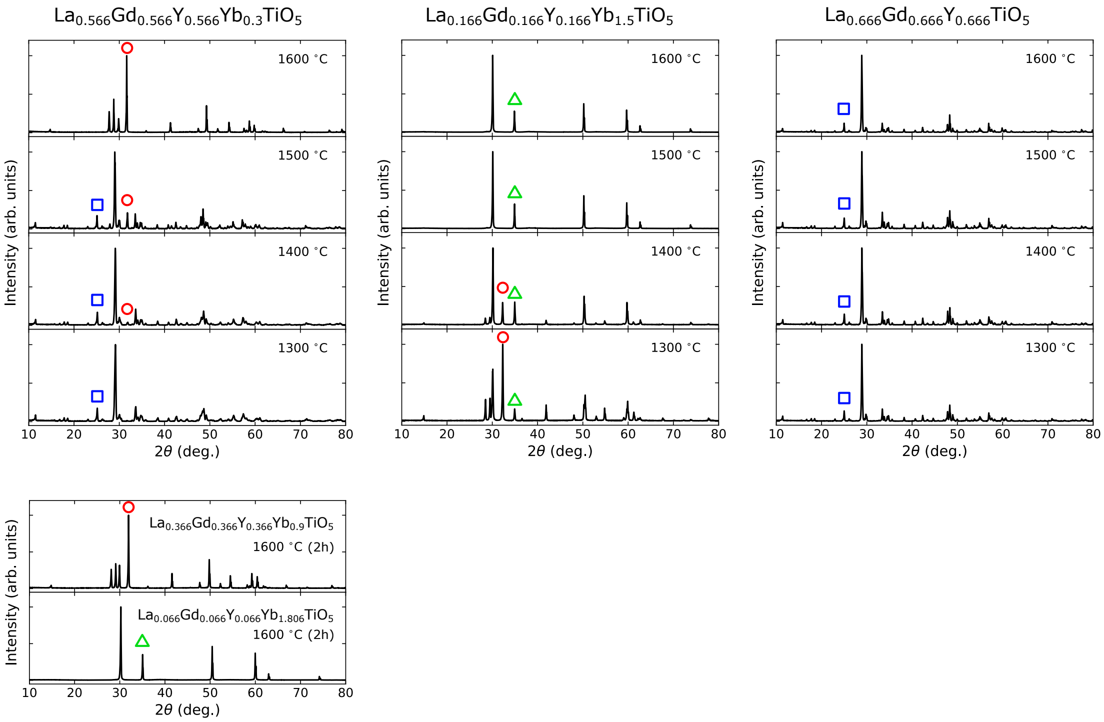

Figure S3. XRD patterns of $La_{(2-x)/3}Gd_{(2-x)/3}Y_{(2-x)/3}Yb_xTiO_5$ (x = 0, 0.3, 0.9, 1.5, 1.8), prepared via thermal treatment for 50 h at each temperature, unless otherwise specified for the treated time. The symbols are the same as those in Figure S2. The identified phases are listed in Table S1.

Table S1. Crystal structure of $La_{(2-x)/3}Gd_{(2-x)/3}Y_{(2-x)/3}Yb_xTiO_5$ (x = 0, 0.3, 0.9, 1.5, 1.8), identified by XRD (Figure S3). Average ionic radius of rare-earth ions with a coordination number of eight, $\langle R^{3+}_{\mathrm{ion}} \rangle$, is expressed in pm.

Composition	$\langle R^{3+}_{\mathrm{ion}} \rangle$	1300 °C	1400 °C	1500 °C	1600 °C
$La_{0.666}Gd_{0.666}Y_{0.666}TiO_5$	107.7	O	O	O	O
$La_{0.566}Gd_{0.566}Y_{0.566}Yb_{0.3}TiO_5$	106.3	O	O + H	O + H	H
$La_{0.366}Gd_{0.366}Y_{0.366}Yb_{0.9}TiO_5$	103.6	–	–	–	H
$La_{0.166}Gd_{0.166}Y_{0.166}Yb_{1.5}TiO_5$	100.8	H + C	H + C	C	C
$La_{0.066}Gd_{0.066}Y_{0.066}Yb_{1.806}TiO_5$	99.4	–	–	–	C

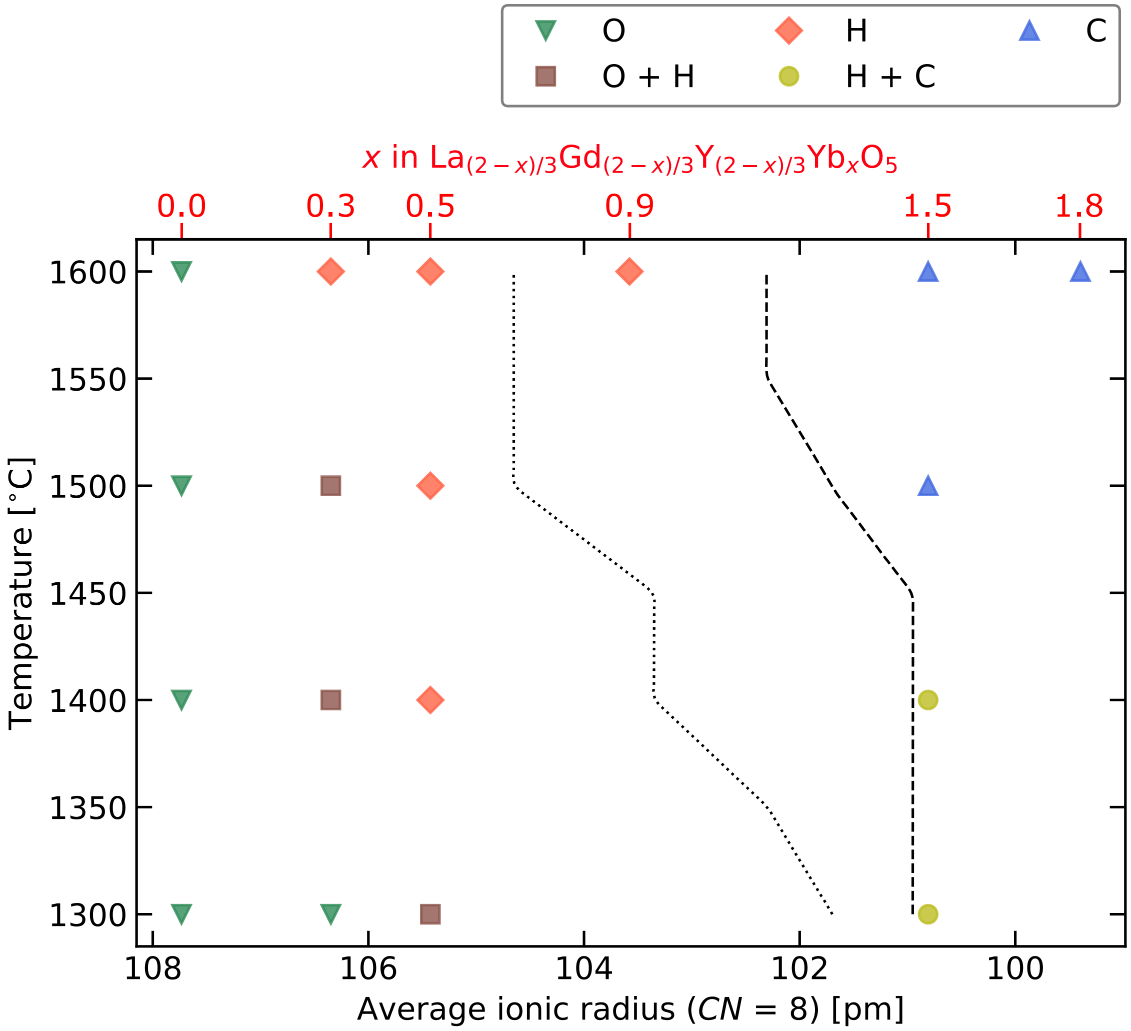

Figure S4. Crystalline-phase map of for $La_{(2-x)/3}Gd_{(2-x)/3}Y_{(2-x)/3}Yb_xTiO_5$ ($x = 0$, 0.3, 0.5, 0.9, 1.5, 1.8) as a function of the average ionic radius with a coordination number of eight. The dotted and dashed lines indicate the phase boundaries between the O and H phases and the H and C phases for $(1R)_2TiO_5$, respectively.

Cubic phase $Dy_{0.5}Y_{0.5}Er_{0.5}Yb_{0.5}TiO_5$

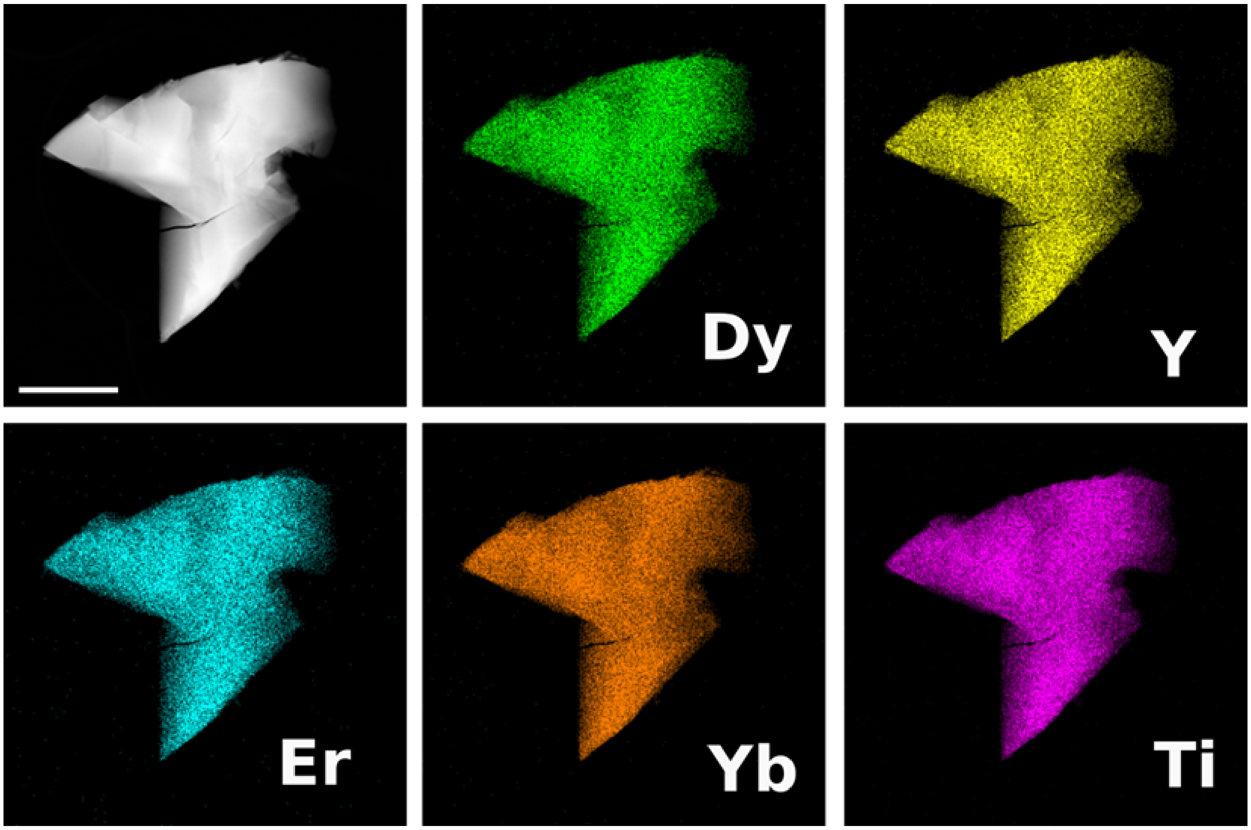

Figure S5. Annular dark-field (ADF) image and energy-dispersive X-ray spectroscopy (EDS) elemental maps of each cation for cubic phase $Dy_{0.5}Y_{0.5}Er_{0.5}Yb_{0.5}TiO_5$ from the [110] zone axis. The scale bar is 0.5 μm. These micrographs verify the compositional homogeneity at the micrometer scale in the sample.

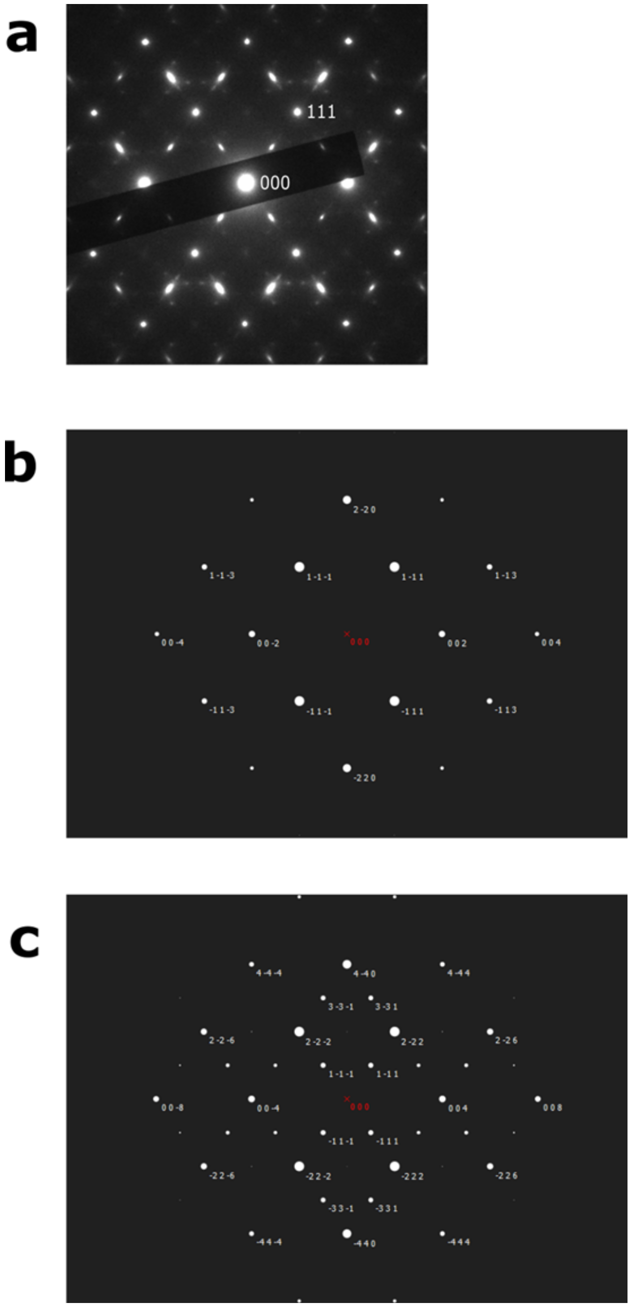

Figure S6. Electron diffraction pattern of cubic phase $Dy_{0.5}Y_{0.5}Er_{0.5}Yb_{0.5}TiO_5$ from the [110] zone axis. a) Experimental pattern with the 111 refection label of the defect-fluorite structure ($Fm\overline{3}m$), which is identical to the Fourier-transformed image (Figure 2b of the main text). b,c) Simulated patterns with the defect-fluorite ($Fm\overline{3}m$) and pyrochlore ($Fd\overline{3}m$) structures, respectively. The experimentally observed spots in the middle of the spots of the defect-fluorite structure are attributed to the pyrochlore-type ordering of cations.

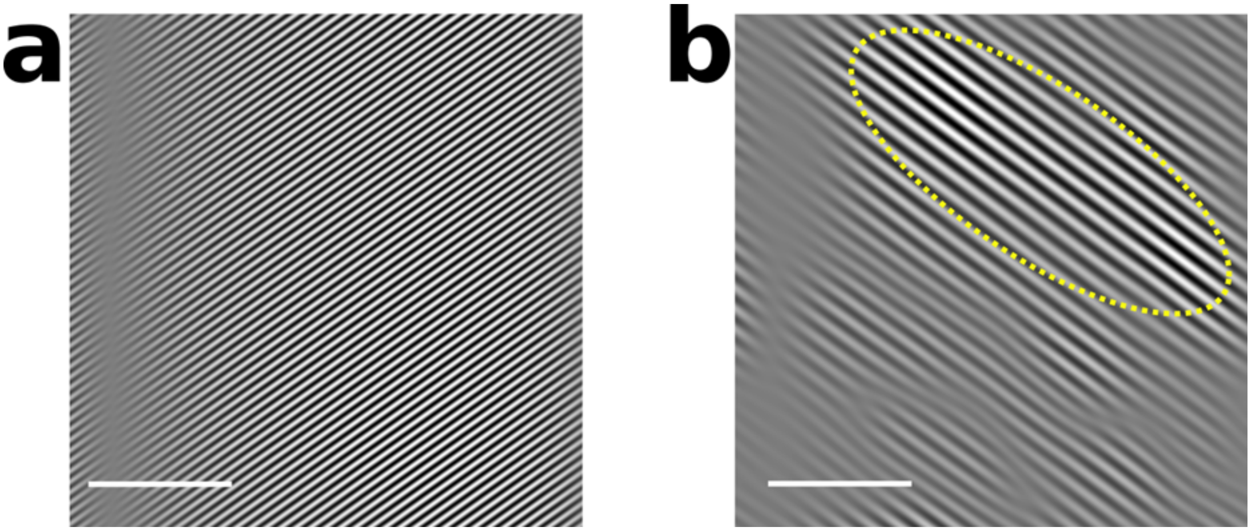

Figure S7. Real-space image via inverse Fourier-transformation of cubic phase $Dy_{0.5}Y_{0.5}Er_{0.5}Yb_{0.5}TiO_5$ from the [110] zone axis. The scale bars indicate 5 nm. a) Image corresponding to the 111 reflection spots of the defect-fluorite structure, indicated by green dashed circles on the Fourier-transformed image (Figure 2b of the main text). b) Real-space image corresponding to the additional spots in the middle of the 111 reflection spots of the defect-fluorite structure, indicated by blue dashed circles on the Fourier-transformed image (Figure 2b of the main text).

Table S2. Crystallographic data from the Rietveld refinement of cubic phase $Dy_{0.5}Y_{0.5}Er_{0.5}Yb_{0.5}TiO_5$ ($Fm\overline{3}m$). The "Wyck." and "Occ." columns indicate the Wyckoff letter and occupancy, respectively.

Atom	Wyck.	x	y	z	B [Å^2]	Occ.
Dy	4a	0	0	0	2.286(13)	0.1627
Y	4a	0	0	0	2.286(13)	0.1668
Er	4a	0	0	0	2.286(13)	0.1643
Yb	4a	0	0	0	2.286(13)	0.1651
Ti	4a	0	0	0	2.286(13)	0.3411
O	8c	0.25	0.25	0.25	6.364(116)	0.8353

[a] R/Ti ratio: 1.9314, a = 5.15121(2) Å, ρ = 6.82 g/cm^3, R_{wp} = 4.79, R_{B} = 3.56, R_{F} = 2.50.

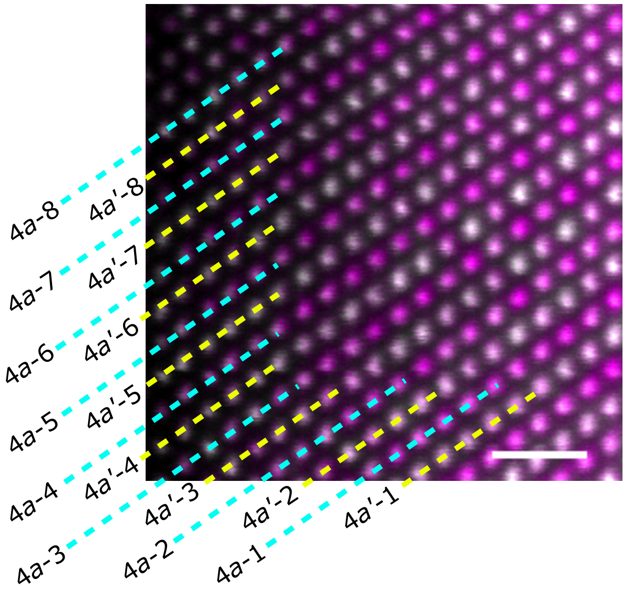

Figure S8. ADF image with an overlapping EDS image of Ti (purple color) from the [110] zone axis for cubic phase $Dy_{0.5}Y_{0.5}Er_{0.5}Yb_{0.5}TiO_5$. The scale bar is 1nm. The blue and yellow dashed lines labeled $4a$ and $4a'$ indicate the two types of lines with Ti-rich and Ti-poor columns, respectively; these are used for analyses of the cation occupancy ratio (Figure 2e of the main text).

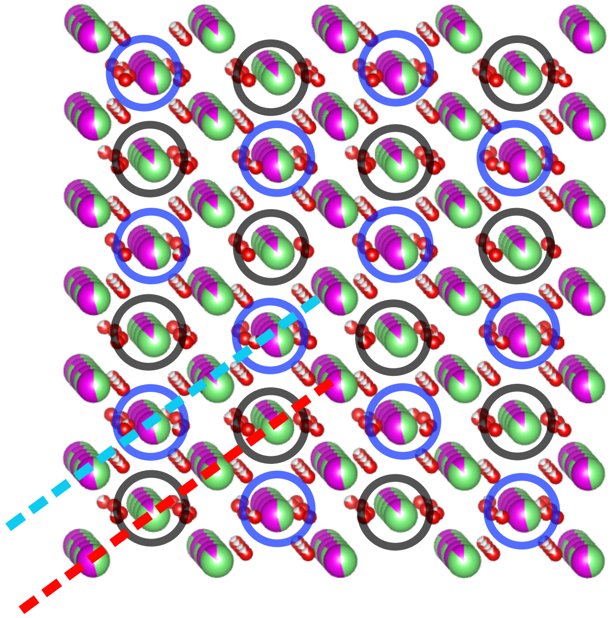

Figure S9. Pyrochlore structure model viewed from the near [110] direction, with a slight tilt for clarity, for cubic phase $Dy_{0.5}Y_{0.5}Er_{0.5}Yb_{0.5}TiO_5$. While oxygen atoms are represented by red spheres with partial occupancy, cations are shown as purple and green spheres, corresponding to Ti and R elements, respectively. The blue and black circles represent Ti-rich and Ti-poor columns, corresponding to the 16c and 16d sites of the pyrochlore structure ($Fd\bar{3}m$), respectively. In each remaining cation column, two sites reside in an alternating manner. The Ti-rich and Ti-poor columns align along the blue and red dashed lines, respectively.

Hexagonal phase Dy_2TiO_5

Table S3. Density of polycrystals of hexagonal phase Dy_2TiO_5 and $Gd_{0.5}Dy_{0.5}Y_{0.5}Yb_{0.5}TiO_5$, in the units of g/cm^3.The reference values for Dy_2TiO_5 exhibit significant scatter.

	Dy_2TiO_5	$Gd_{0.5}Dy_{0.5}Y_{0.5}Yb_{0.5}TiO_5$
Previous model	6.58	6.08
Revised model (Stuffed model (B))	7.30	6.79
Exp. (This work)	7.26	6.80
Exp. (References)	6.65[a], 7.04[b], 7.1-7.2[c]	

[a] Ref. [1].
[b] Ref. [2].
[c] Ref. [3].

Table S4. Crystallographic data from the Rietveld refinement of hexagonal phase Dy_2TiO_5 ($P6_3/mmc$). The "Wyck." and "Occ." columns indicate the Wyckoff letter and occupancy, respectively.

Atom	Wyck.	x	y	z	B [Å^2]	Occ.
Dy1	4e	0	0	0.02210(6)	0.524(13)	0.4895(6)
Ti1	4e	0	0	0.02210(6)	0.524(13)	0.0105(-)
Dy2	2c	0.3333	0.6667	0.25	3.112(39)	0.3358(-)
Ti2	2c	0.3333	0.6667	0.25	3.112(39)	0.6642(-)
O1	4f	0.3333	0.6667	0.08129(35)	2.372(119)	1
O2	2b	0	0	0.25	6.673(425)	0.8600(163)
O3	2d	0.3333	0.6667	0.75	13.327(1273)	0.4826(-)

[a] Dy/Ti ratio: 1.9189, $a = b = 3.63319(2)$ Å, $c = 11.93720(8)$ Å, $\rho = 7.30$ g/cm^3, $R_{\mathrm{wp}} = 4.82$, $R_{\mathrm{B}} = 5.67$, $R_{\mathrm{F}} = 3.07$.

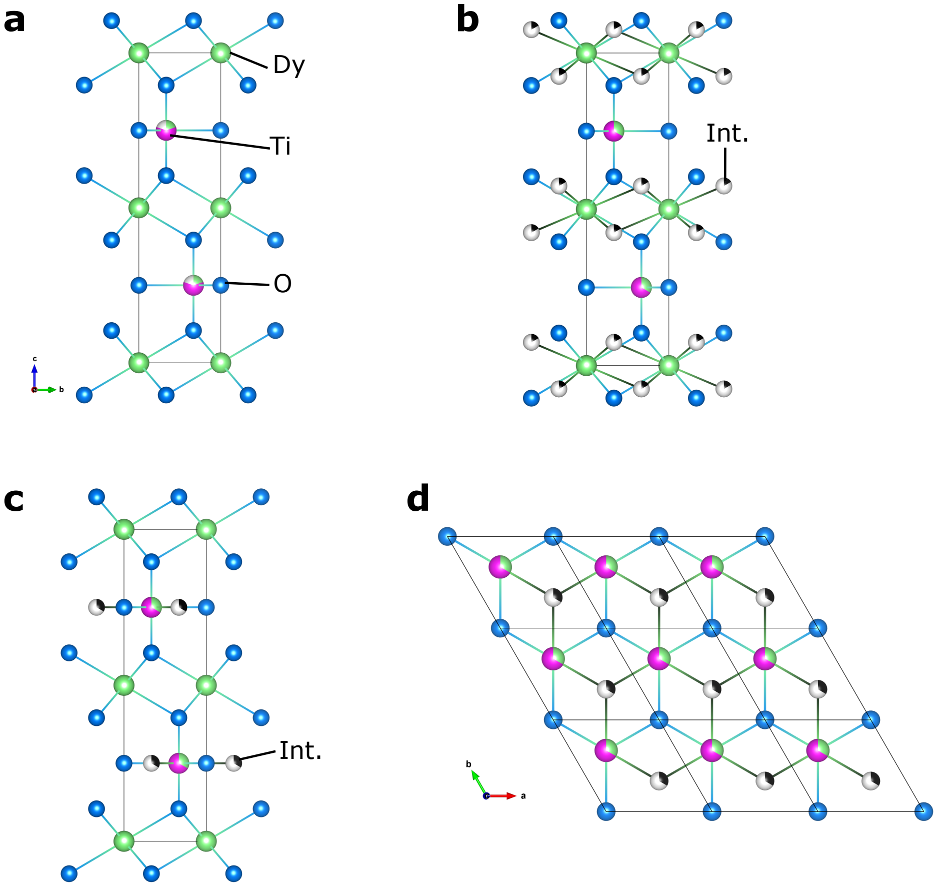

Figure S10. Structural model of hexagonal phase Dy_2TiO_5 with $P6_3/mmc$ symmetry. a) The previous model,[4] which is composed of the Dy-occupying 2a and Ti-rich 2c sites for cations. b) Stuffed model (A). c) Stuffed model (B). d) Stuffed model (B) viewed from the c axis. In the stuffed models, cation vacancies at the 2c site are filled by cations under the constraint that the Dy:Ti ratio is 2:1. To maintain stoichiometry, oxygen atoms are added into interstitial sites which are near the 2a site for the stuffed model (A) and located on the layer containing the 2c site for the stuffed model (B).

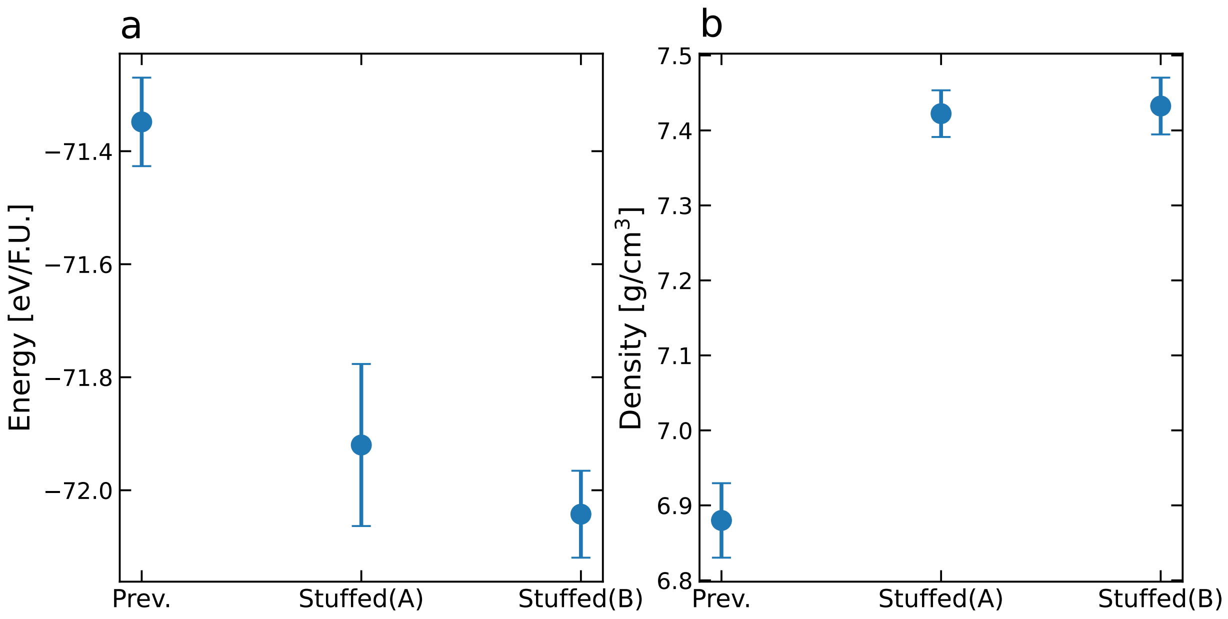

Figure S11. Calculated results for hexagonal phase Dy_2TiO_5. a) Energy. b) Density. Here, the data for 20 SQS models are averaged, and the error bars indicate the standard deviation. The energy of the previous model is clearly greater than that of the stuffed models, and the stuffed model (B) tends to be more stable than the model (A), as shown in (a). The densities of the stuffed models are similar to each other, with a higher value than the previous model by approximately 0.55 g/cm^3, as shown in (b). This difference is similar to that between the experimental densities determined via the refinement with the previous model and the measured value (0.68 g/cm^3).

Hexagonal phase $Gd_{0.5}Dy_{0.5}Y_{0.5}Yb_{0.5}TiO_5$

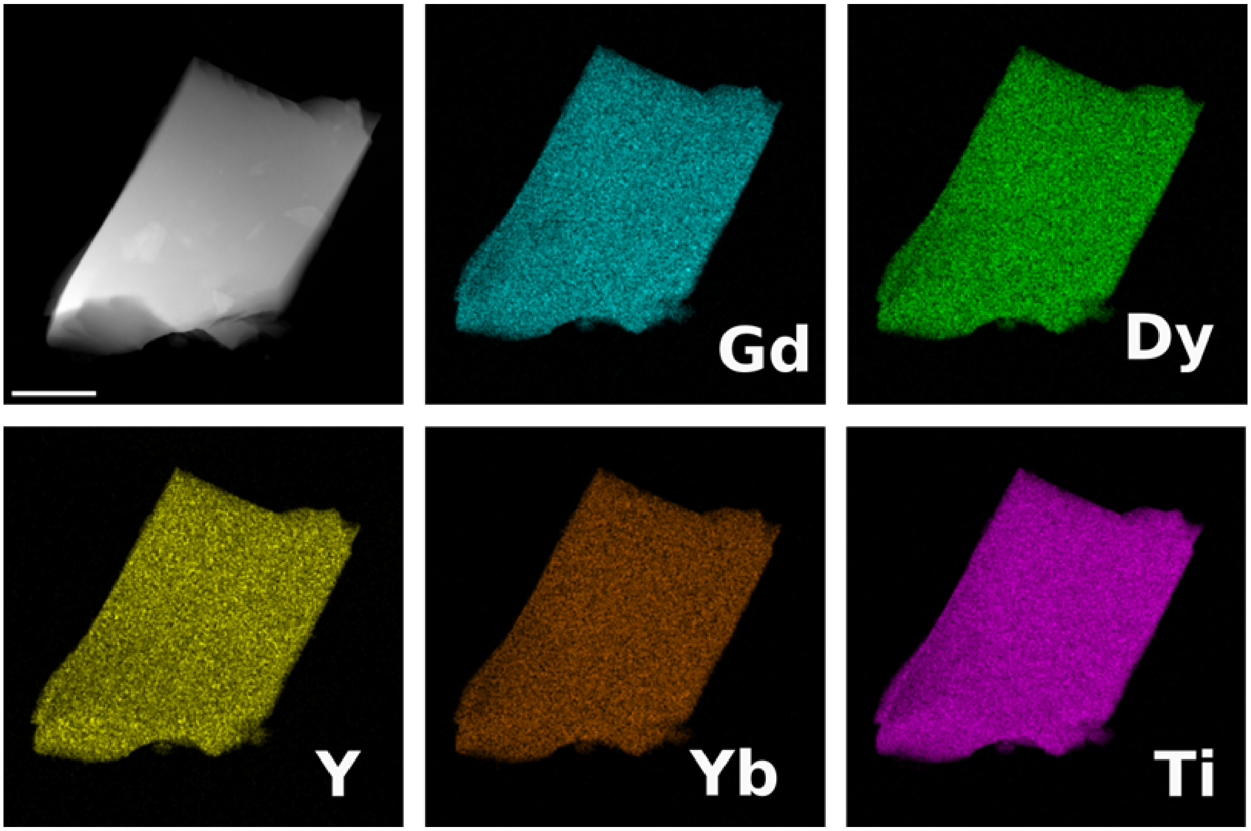

Figure S12. ADF image and EDS elemental maps of each cation for hexagonal phase $Gd_{0.5}Dy_{0.5}Y_{0.5}Yb_{0.5}TiO_5$ from the [100] zone axis. The scale bar is 0.25 μm. These micrographs verify the compositional homogeneity of the sample at the micrometer scale.

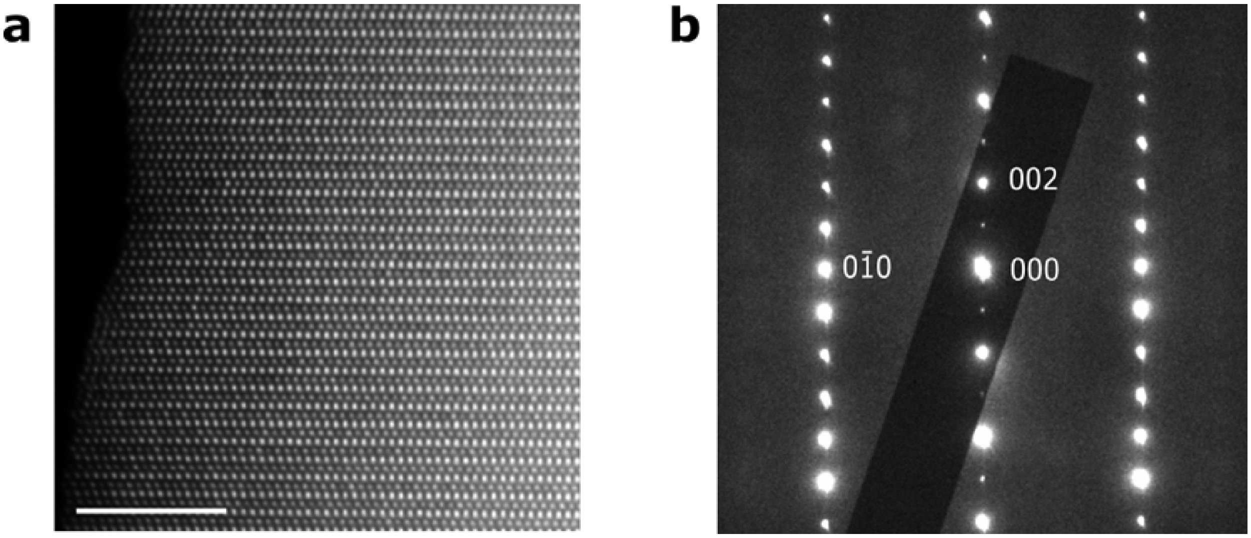

Figure S13. Electron diffraction characterization of hexagonal phase $Gd_{0.5}Dy_{0.5}Y_{0.5}Yb_{0.5}TiO_5$. a) ADF image from the [100] zone axis; here, layered structures are clearly observed. The scale bar is 5 nm. b) Electron diffraction pattern corresponding to $P6_3/mmc$ symmetry (Table S5).

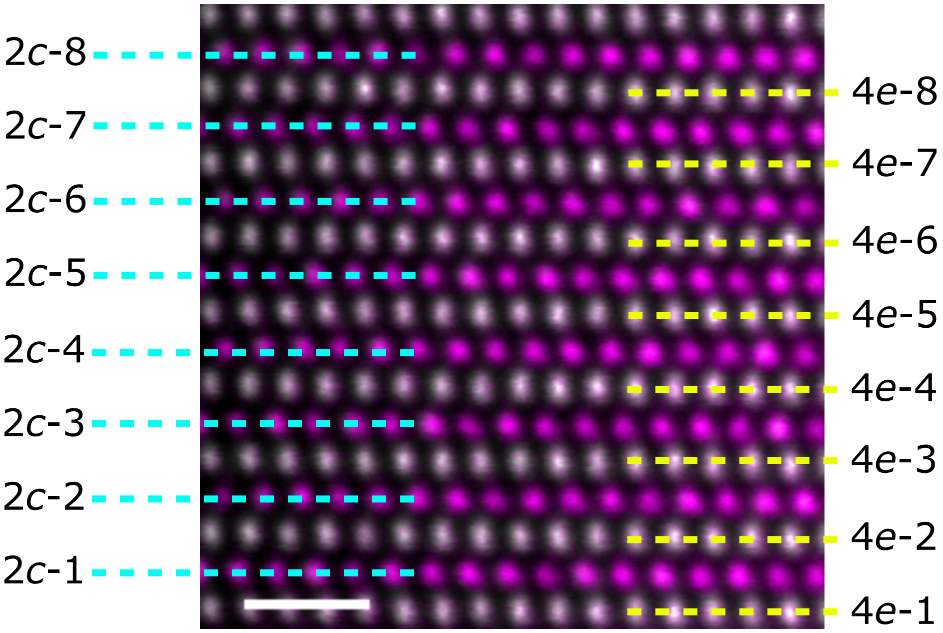

Figure S14. ADF image with an overlapping EDS image of Ti (purple color) from the [100] zone axis for the hexagonal phase $Gd_{0.5}Dy_{0.5}Y_{0.5}Yb_{0.5}TiO_5$. The scale is 1 nm. The blue and yellow dashed lines labeled $2c$ and $4e$ include the Ti-rich and Ti-poor sites, respectively; these are used for analyses of the cation occupancy ratio (Figure 3e of the main text)

Table S5. Crystallographic data from the Rietveld refinement of hexagonal phase $Gd_{0.5}Dy_{0.5}Y_{0.5}Yb_{0.5}TiO_5$ ($P6_3/mmc$). The "Wyck." and "Occ." columns indcate the Wyckoff letter and occupancy respectively.

Atom	Wyck.	x	y	z	B [Å^2]	Occ.
Yb1	4e	0	0	0.02135(10)	0.296(20)	0.0908
Y1	4e	0	0	0.02135(10)	0.296(20)	0.1068
Gd1	4e	0	0	0.02135(10)	0.296(20)	0.1252
Dy1	4e	0	0	0.02135(10)	0.296(20)	0.1070
Ti1	4e	0	0	0.02135(10)	0.296(20)	0.0702
Yb2	2c	0.3333	0.6667	0.25	5.5000(52)	0.1481
Y2	2c	0.3333	0.6667	0.25	5.5000(52)	0.1140
Gd2	2c	0.3333	0.6667	0.25	5.5000(52)	0.0850
Dy2	2c	0.3333	0.6667	0.25	5.5000(52)	0.1077
Ti2	2c	0.3333	0.6667	0.25	5.5000(52)	0.5452
O1	4f	0.3333	0.6667	0.07242(49)	4.616(160)	1
O2	2b	0	0	0.25	4.616(160)	0.8296(80)
O3	2d	0.3333	0.6667	0.75	4.616(160)	0.5132(-)

[a] R/Ti ratio: 1.9171, $a = b = 3.62509(4)$ Å, $c = 11.91892(12)$ Å, $\rho = 6.79$ g/cm^3, $R_{wp} = 5.69$, $R_B = 5.83$, $R_F = 3.18$.

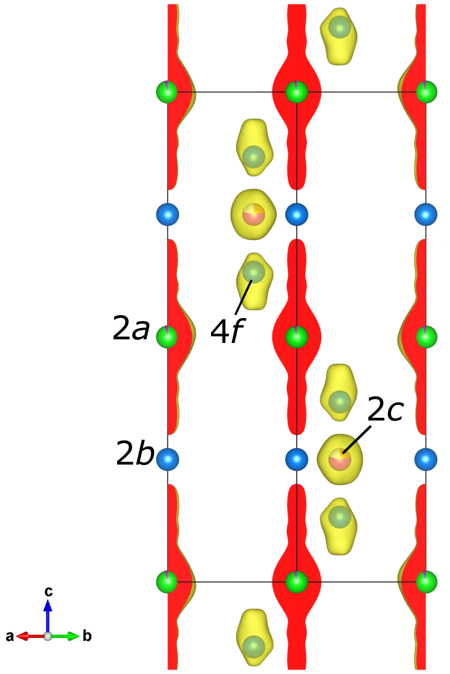

Figure S15. Electron density map generated using the maximum entropy method (MEM) for hexagonal phase $Gd_{0.5}Dy_{0.5}Y_{0.5}Yb_{0.5}TiO_5$ with the previous structure model.

Orthorhombic phase $La_{0.5}Nd_{0.5}Gd_{0.5}Dy_{0.5}TiO_5$

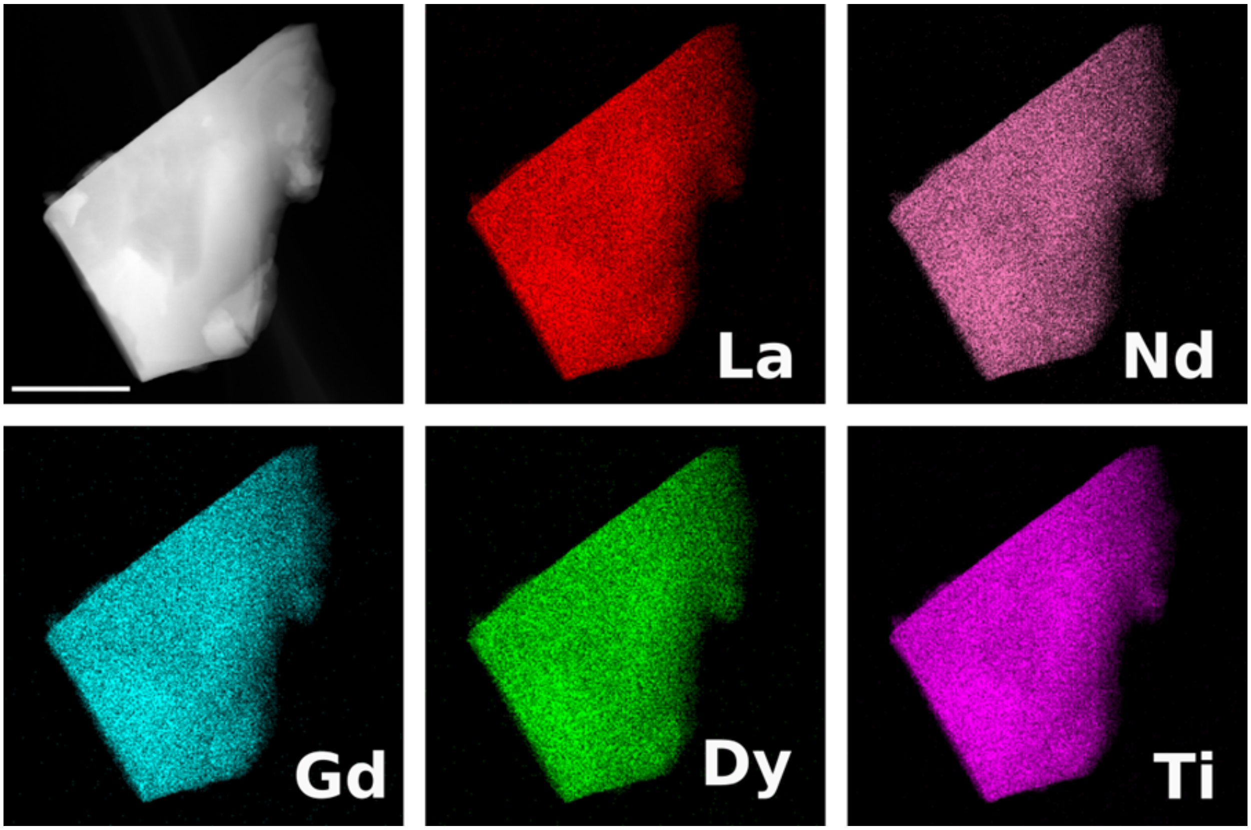

Figure S16. ADF image and EDS elemental maps of each cation for orthorhombic phase $La_{0.5}Nd_{0.5}Gd_{0.5}Dy_{0.5}TiO_5$ from the [010] zone axis. The scale bar is 0.2 μm. These micrographs verify the compositional homogeneity of the samples at the micrometer scale.

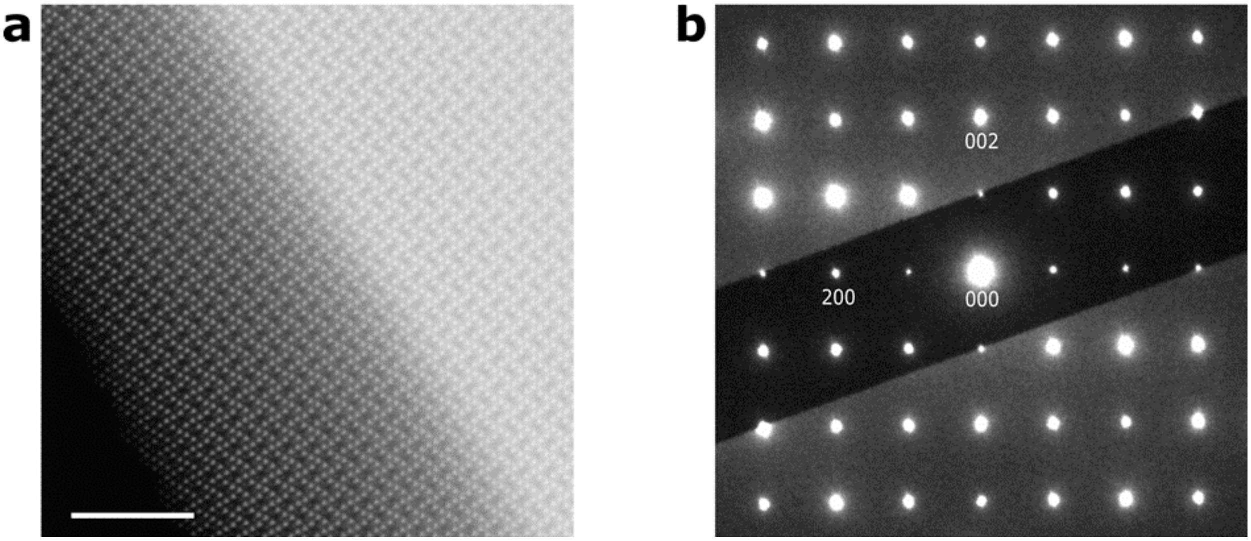

Figure S17. Electron diffraction characterization of orthorhombic phase $La_{0.5}Nd_{0.5}Gd_{0.5}Dy_{0.5}TiO_5$. a) ADF image from the [010] zone axis. The scale bar is 5 nm. b) Electron diffraction pattern corresponding to *Pnma* symmetry (Table S6).

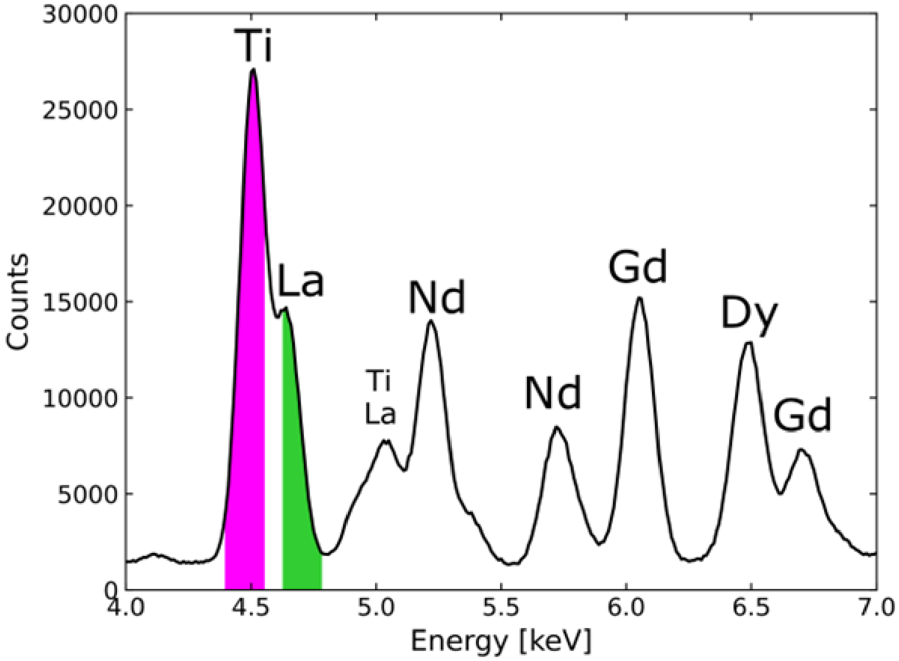

Figure S18. EDS spectra for separately mapping Ti and La in orthorhombic phase $La_{0.5}Nd_{0.5}Gd_{0.5}Dy_{0.5}TiO_5$. The purple and green areas indicate the energy ranges for the mapping of Ti and La, respectively.

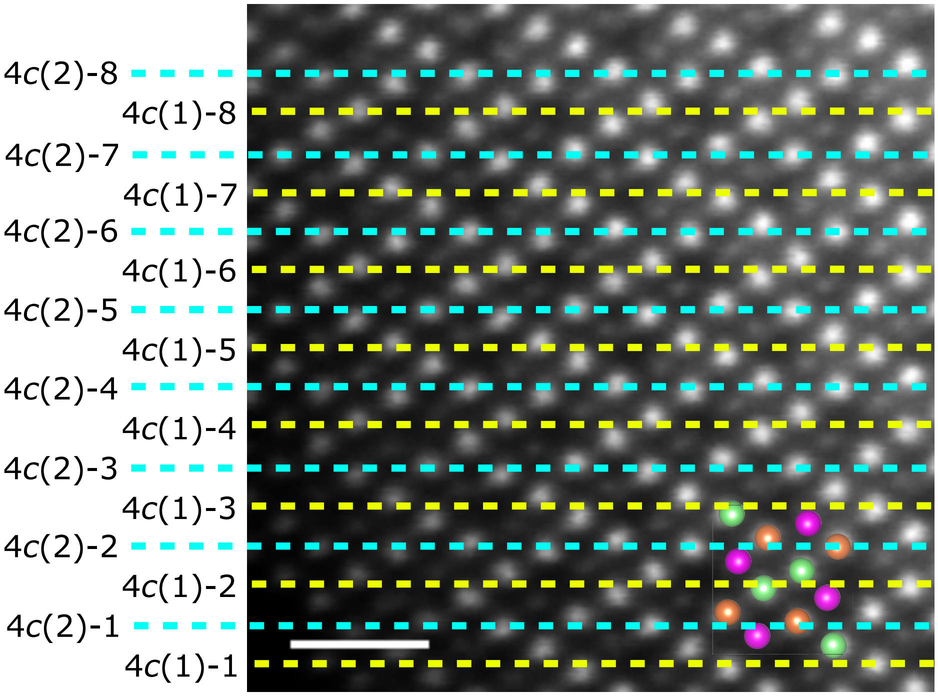

Figure S19. ADF image from the [010] zone axis for orthorhombic phase $La_{0.5}Nd_{0.5}Gd_{0.5}Dy_{0.5}TiO_5$. The scale bar is 1 nm. The yellow and blue dashed lines correspond to the lines including the $4c(1)$ and $4c(2)$ sites, respectively; these are used for the analyses of the cation occupancy ratio (Figure 4b of the main text).

Table S6. Crystallographic data from the Rietveld refinement of the orthorhombic phase $La_{0.5}Nd_{0.5}Gd_{0.5}Dy_{0.5}TiO_5$ (*Pnma*). The "Wyck." and "Occ." columns indicate the Wyckoff letter and occupancy, respectively.

Atom	Wyck.	x	y	z	B [Å^2]	Occ.
La1	4c(1)	0.13646(6)	0.25	0.05910(5)	0.351(12)	0.2464
Nd1	4c(1)	0.13646(6)	0.25	0.05910(5)	0.351(12)	0.2471
Gd1	4c(1)	0.13646(6)	0.25	0.05910(5)	0.351(12)	0.2468
Dy1	4c(1)	0.13646(6)	0.25	0.05910(5)	0.351(12)	0.24
Ti1	4c(1)	0.13646(6)	0.25	0.05910(5)	0.351(12)	0.0148
La2	4c(2)	0.39408(6)	0.75	0.22006(6)	0.384(13)	0.2464
Nd2	4c(2)	0.39408(6)	0.75	0.22006(6)	0.384(13)	0.2471
Gd2	4c(2)	0.39408(6)	0.75	0.22006(6)	0.384(13)	0.2468
Dy2	4c(2)	0.39408(6)	0.75	0.22006(6)	0.384(13)	0.24
Ti2	4c(2)	0.39408(6)	0.75	0.22006(6)	0.384(13)	0.0148
Ti3	4c(3)	0.18198(18)	0.25	0.37753(20)	0.324(35)	1
O1	4c(4)	0.22255(60)	0.25	0.53650(60)	0.570(154)	1
O2	4c(5)	0.49201(61)	0.25	0.60271(64)	0.577(146)	1
O3	4c(6)	0.26455(59)	0.25	0.87811(65)	0.689(151)	1
O4	4c(7)	0.02164(75)	0.25	0.34328(67)	2.390(211)	1
O5	4c(8)	0.26609(69)	0.25	0.23315(64)	0.944(168)	1

[a] R/Ti ratio: 1.9043, a = 10.59319(7) Å, b = 3.80158(2) Å, c = 11.30409(7) Å, ρ = 6.20 g/cm^3, R_{wp} = 5.26, R_{B} = 2.22, R_{F} = 1.07.

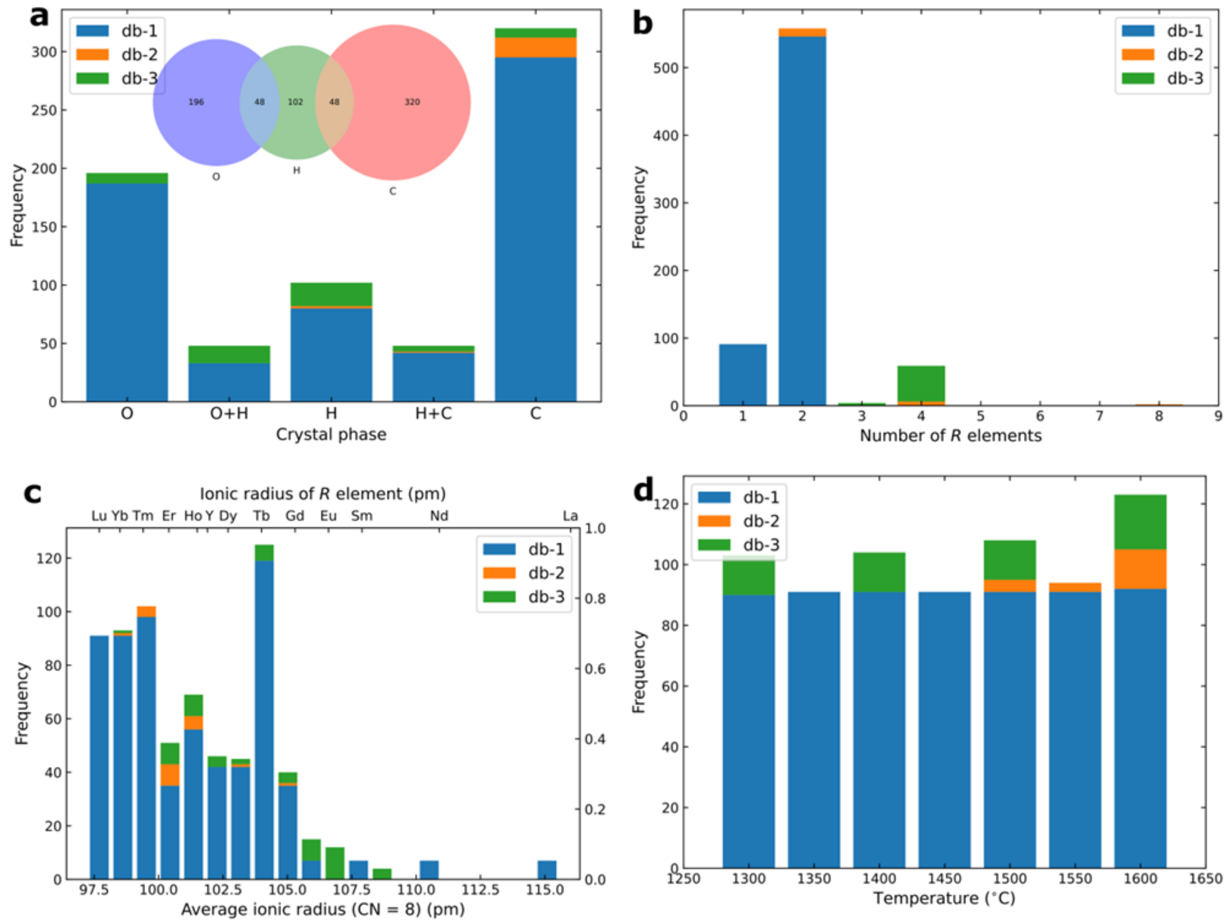

Figure S20. Collected data of the R_2TiO_5 crystalline phases with histogram representation. The database is categorized into three types: db-1 and db-2 are extracted from the Phase Equilibria Diagrams Database[5] and from the literature by Augtherson et al., respectively (Table S7), whereas db-3 is obtained in our study (Table 1 of the main text and Table S1). a) Distribution of the crystalline phases, with the Venn diagram representation in the inset. b) Distribution of the number of R elements. c) Distribution of the average ionic radius of R^{3+} ions with a coordination number of eight. d) Distribution of the temperature.

Table S7. Collected data from the R_2TiO_5 crystalline phases from the database and literature. Database kind 1 (db-1) is collected from the Phase Equilibria (PE) database provided by the American Ceramic Society and the National Institute of Standards and Technology[5] and database kind 2 (db-2) is extracted from a series of papers by Aughterson et al. N_R and N_{data} represent the number of rare-earth elements and the number of collected data for each composition, respectively. The "Reference" column contains literature cited for the determination of the crystalline phases.

Database	N_R	R in R_2TiO_5	PE figure-id	Reference	N_{data}
db-1	1	La	2373	[6, 7]	7
	1	Nd	15390	[8]	7
	1	Sm	9334	[9]	7
	1	Eu	91-032	[10]	7
	1	Gd	2368	[4, 11]	7
	1	Tb	6498	[12]	7
	1	Dy	9290	[13]	7
	1	Ho	9303	[14, 15]	7
	1	Er	6467	[16]	7
	1	Tm	9340	[17]	7
	1	Yb	6508	[18]	7
	1	Lu	6487	[19]	7
	1	Y	6502	[20]	7
	2	Er Lu	9659	[21]	133
	2	Gd Lu	9679	[21]	133
	2	Tb Lu	9687	[21]	133
	2	Gd Tb	12283	[22]	133
	2	La Lu	12284	[22]	14
				Total:	637
db-2	2	Tb Yb		[23]	4
	2	Ho Yb		[15]	5
	4	Sm Gd Dy Yb		[24]	6
	8	La Sm Tb Dy Gd Er Ho Yb		[24]	2
	2	Sm Yb		[25]	3[a]
				Total:	20

[a] These data points are the outliers on the phase map (Figure S21); thus they are omitted.

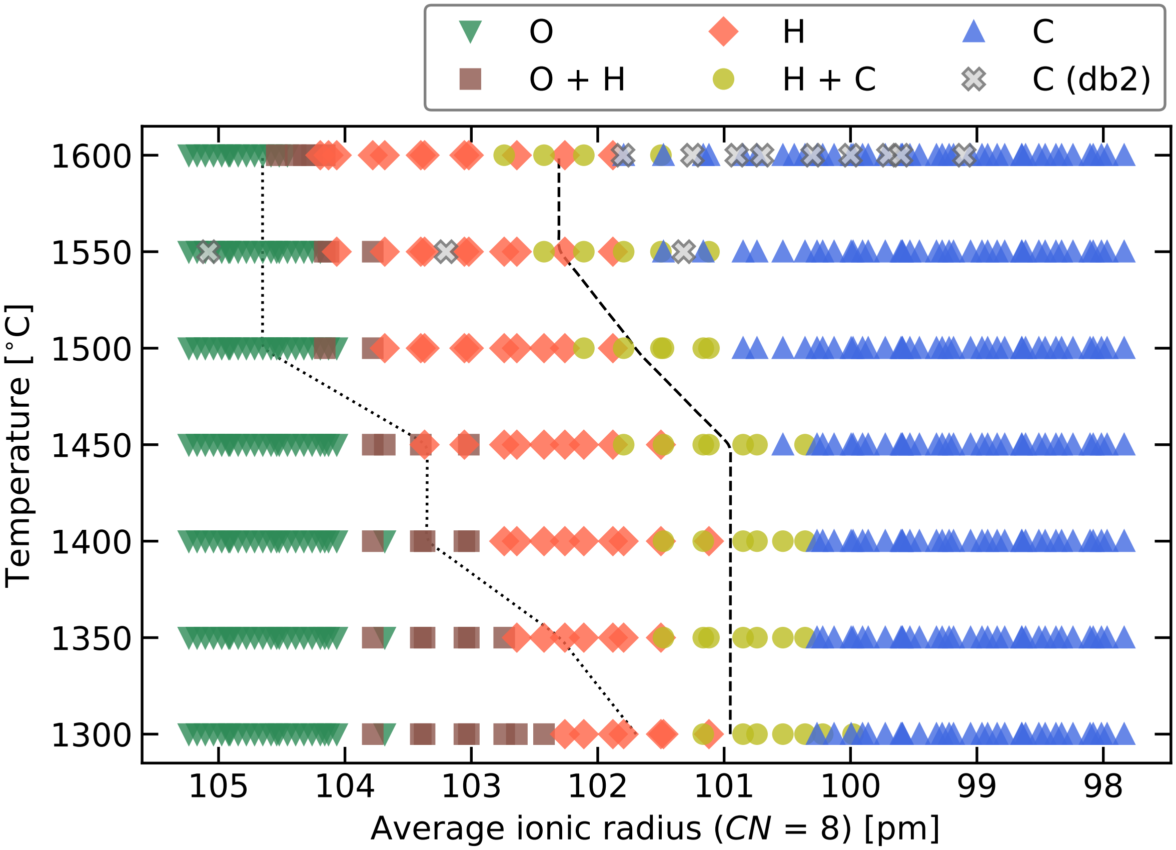

Figure S21. Collected crystalline phase of R_2TiO_5 with two R elements as a function of the average ionic radius with a coordination number of eight (Table S7). The cross symbols represent the db-2 data with the cubic phase; here, few data of the composition with Sm and Yb[25] are distributed in the outlier positions (Table S7). The remaining data belong to db-1.

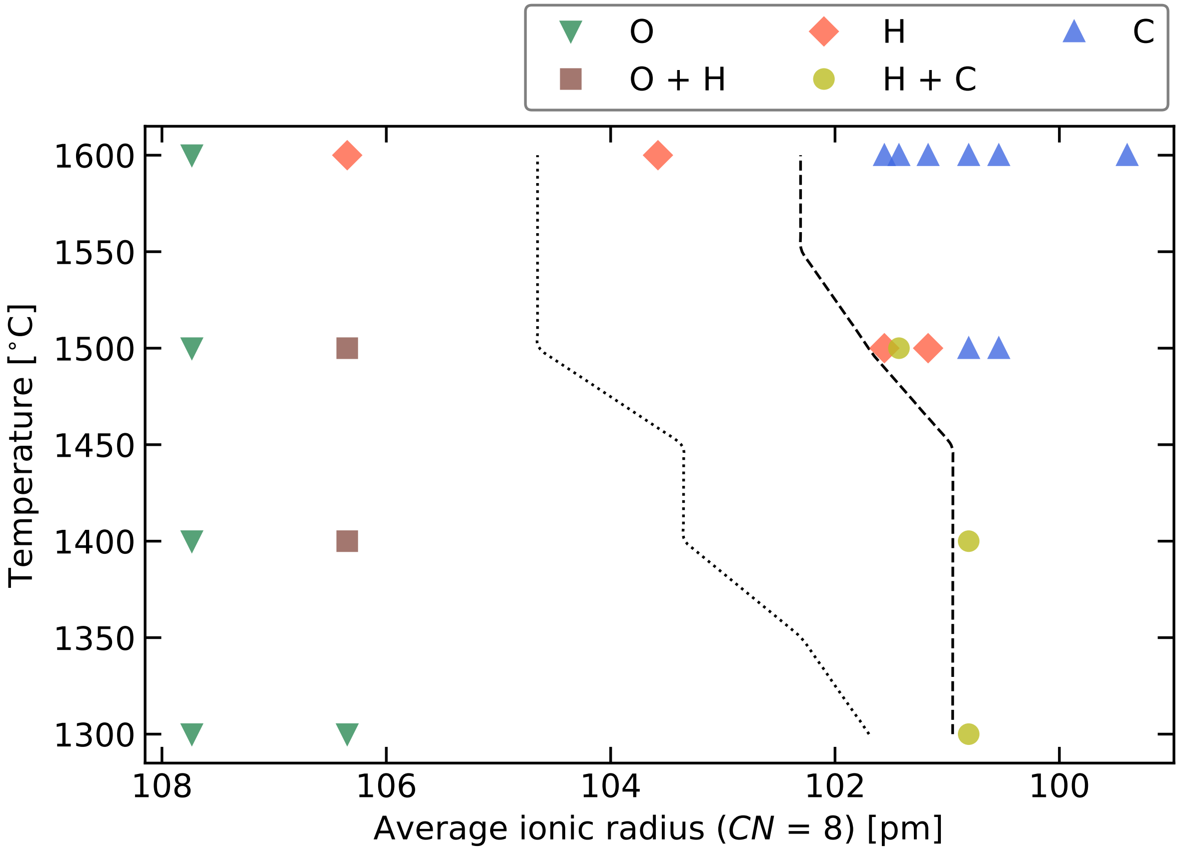

Figure S22. Collected crystalline phase of R_2TiO$_5$ with four and eight R elements belonging to db-2 as a function of the average ionic radius with a coordination number of eight (Table S7).

Supporting Note 1

Here, elemental descriptors for generating features, that are fed into the classifier, are summarized. Two types of elemental features are considered; (1) those provided in the Mendeleev library[26] and (2) those extracted for rare-earth sesquioxide (R_2O_3) from the literature. The feature table is generated via operations for each elemental descriptor using a weighted mean, a weighed standard deviation, and maximum, and minimum values.

(1) Elemental descriptors from the Mendeleev library [26]

Here, we list the descriptor names provided by the library; these are used in the classification scheme (for details, see Ref. [26]): `atomic_volume`, `density`, `dipole_polarizability`, `evaporation_heat`, `specific_heat_capacity`, `vdw_radius`, `covalent_radius_cordero`, `covalent_radius_pyykko`, `en_pauling`, `heat_of_formation`, `vdw_radius_uff`, `vdw_radius_mm3`, `en_ghosh`, `vdw_radius_alvarez`, `c6_gb`, `atomic_radius_rahm`, `covalent_radius_pyykko_double`, `dipole_polarizability_unc`, `glawe_number`, `molar_heat_capacity`, `Rion_3VI`, `Rion_3VIII`, `Rion_3IX`, `ionization_energy_1`, `ionization_energy_2`, `ionization_energy_3`.

(2) Elemental descriptors from R_2O_3 data in the literature

From the literature, we extracted the following elemental descriptors:

- `R2O3_delH0`: Standard enthalpy of formation [27]
- `R2O3_S0`: Standard entropy [27]
- `R2O3_Tm`: Melting point [27]
- `R2O3_relVolm`: Relative variation in molar volume at fusion [27]
- `R2O3_VolA`: Molar volume of A-type sesquioxide including estimated values [27]
- `R2O3_VolB`: Molar volume of B-type sesquioxide including estimated values [27]
- `R2O3_VolC`: Molar volume of C-type sesquioxide including estimated values [27]
- `R2O3_ELatt`: Lattice energy from theoretical calculations [28, 29]
- `GT`: Gibbs energy at a given temperature, calculated from a polynomial equation and reported coefficients [27]
- `CpT`: Heat capacity at a given temperature, calculated from a polynomial equation and reported coefficients [30] (for Y_2O_3, extracted from Ref. [31])

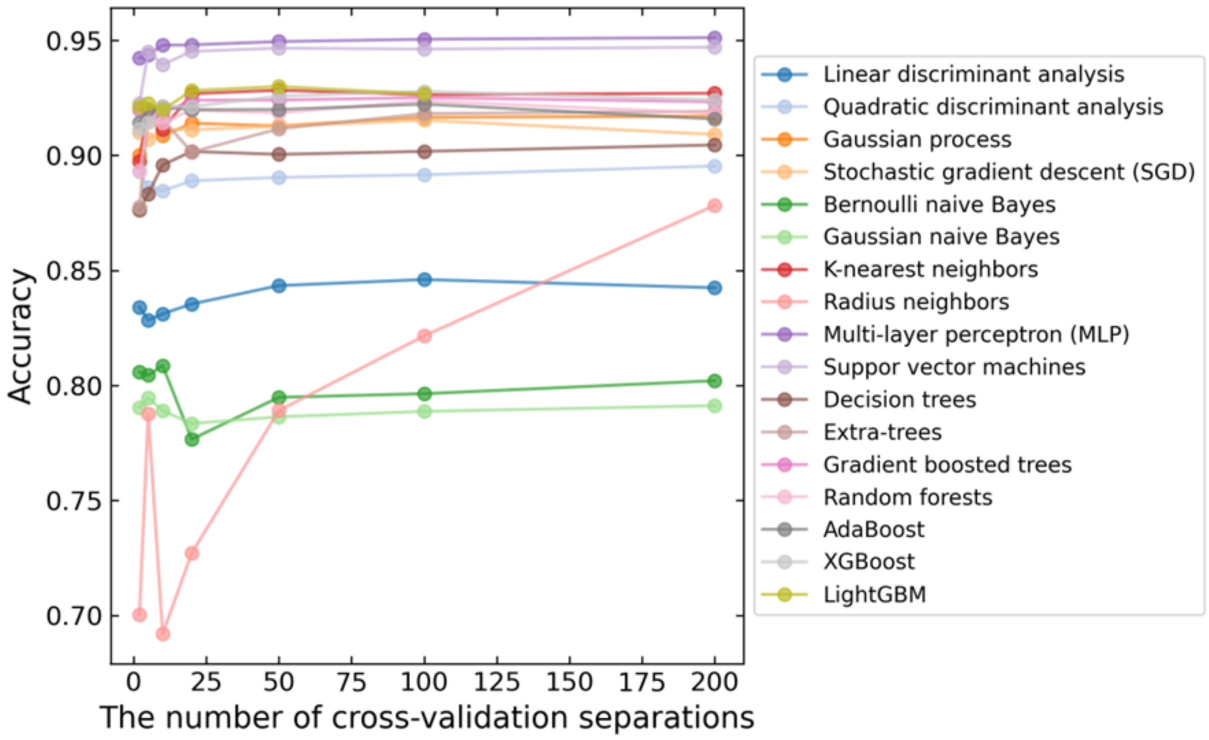

Figure S23. Assessment of classification schemes via cross validation (CV), as a function of the number of CV separations. Several classification models achieve accuracy scores greater than 0.9.

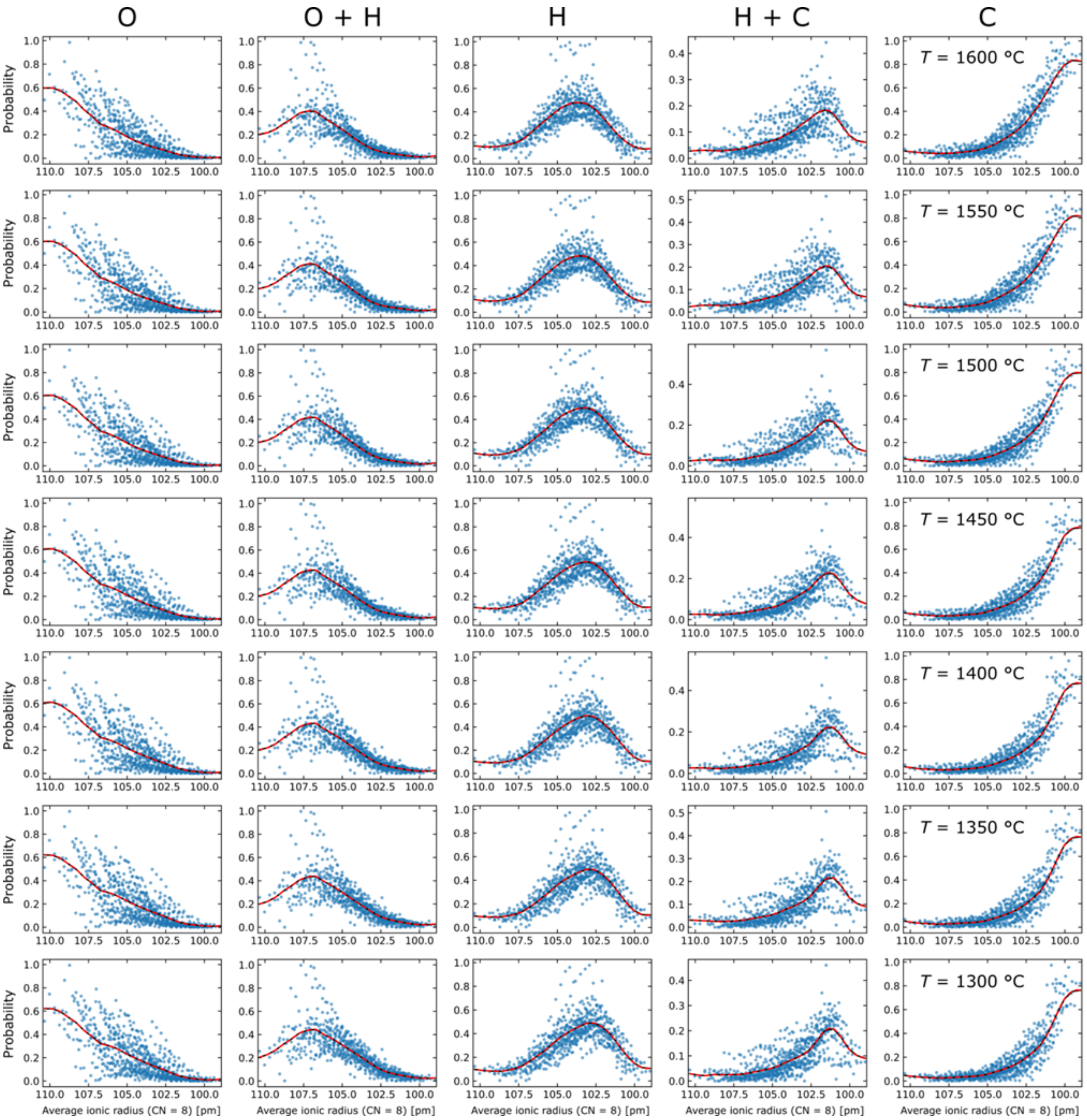

Figure S24. Distribution of the probabilities predicted by the extra-tree classifier for each crystalline phase of R_2TiO_5 with equimolar four R elements as a function of the average ionic radius with a coordination number of eight. The seven rows from bottom to top show the data at temperatures from 1300 to 1600 °C at 50 °C intervals. The five columns from left to right represent the O, O + H, H, H + C, and C phases, respectively. The red lines indicate the average probabilities with a width of 0.5 pm on the vertical axis.

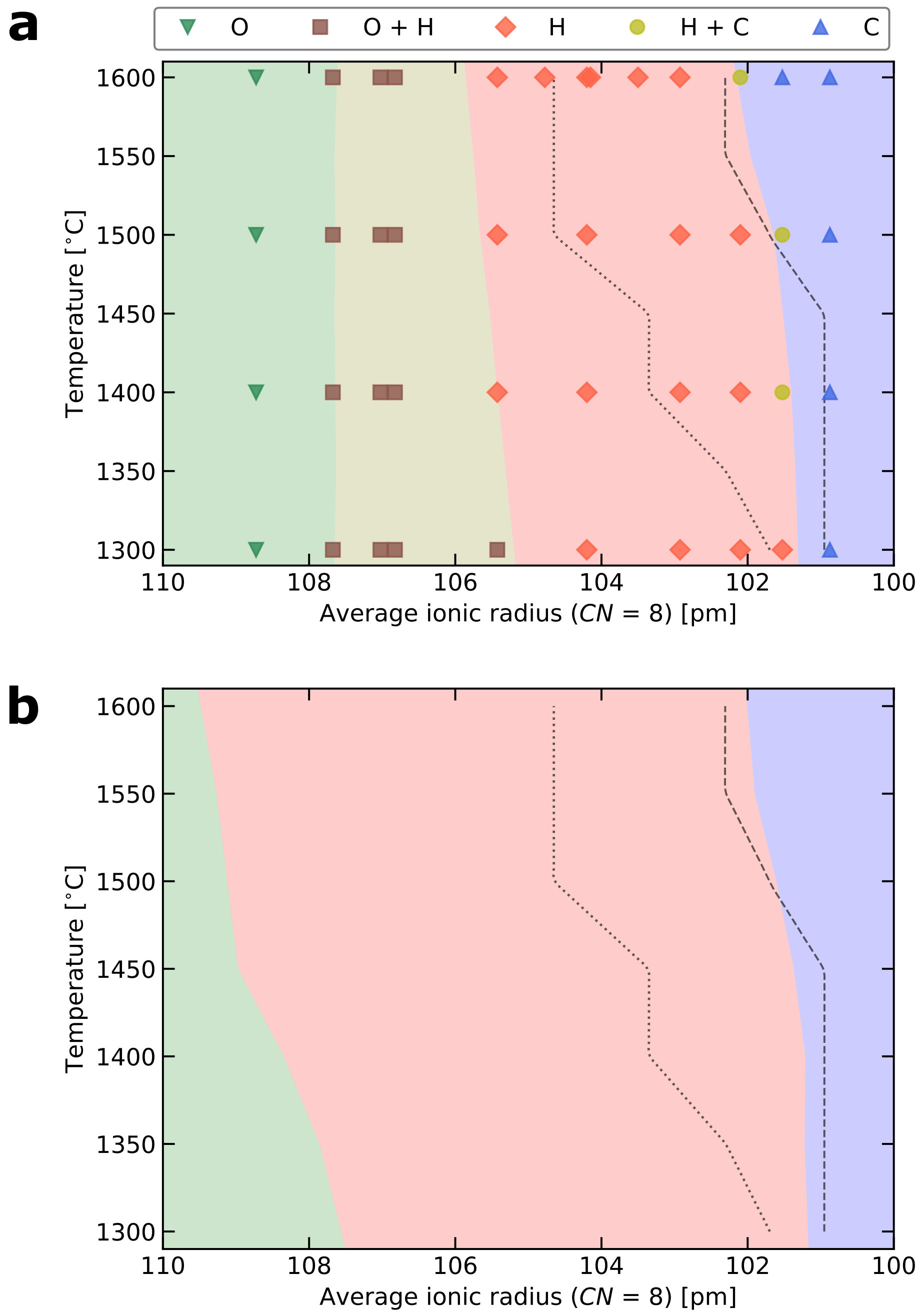

Figure S25. Crystalline-phase map generated by the extra-tree classifier for R_2TiO_5 with equimolar four R elements as a function of the average ionic radius with a coordination number of eight. a) Case using the full training data for classifier training. b) Case using only the db-1 data for the classifier training.

[1] R. D. Aughterson, N. J. Zaluzec, and G. R. Lumpkin, Synthesis and ion-irradiation tolerance of the Dy_2TiO_5 polymorphs, Acta Mater. **204**, 116518 (2021).

[2] C.-H. Jung, C.-J. Kim, and S.-J. Lee, Synthesis and sintering studies on Dy_2TiO_5 prepared by polymer carrier chemical process, J. Nucl. Mater. **354**, 137 (2006).

[3] V. S. Krasnorutskii, S. Y. Saenko, N. N. Belash, I. A. Chernov, A. E. Surkov, N. D. Rybalchenko, and F. V. Belkin, Hot pressing of dysprosium hafnate and titanate pellets, Powder Metall. Met. Ceram. **50**, 708 (2012).

[4] Y. F. Shepelev and M. A. Petrova, Crystal structure of Ln_2TiO_5 (Ln = Gd, Dy) polymorphs, Inorg. Mater. (Engl. Transl.) **44**, 1496 (2008).

[5] The American Ceramic Society and the National Institute of Standards and Technology, Phase equilibria diagrams database version 5.0 (NIST standard reference database 31) (2022).

[6] J. B. MacChesney and H. A. Sauer, The system La_2O_3–TiO_2; phase equilibria and electrical properties, J. Am. Ceram. Soc. **45**, 416 (1962).

[7] S. Hayun and A. Navrotsky, Formation enthalpies and heat capacities of rare earth titanates: RE_2TiO_5 (RE=La, Nd, and Gd), J. Solid State Chem. **187**, 70 (2012).

[8] W. P. Gong and R. Zhang, Phase relationship in the TiO_2–Nd_2O_3 pseudo-binary system, J. Alloys Compd. **548**, 216 (2013).

[9] G. V. Shamrai, R. L. Magunov, and I. P. Kovalevskaya, The system Sm_2O_3-TiO_2, Inorg. Mater. (Engl. Transl.) **22**, 1695 (1986).

[10] B. Iwasaki, Phase equilibria in europium(III) oxide-titanium(IV) oxide system at temperatures from 900 to 1400 °C, Bull. Chem. Soc. Jpn. **51**, 3323 (1978).

[11] J. L. Waring and S. J. Schneider, Phase equilibrium relationships in the system Gd_2O_3–TiO_2, J. Res. Natl. Bur. Stand., Sect. A **69**, 255 (1965).

[12] L. G. Shcherbakova, V. B. Glushkova, K. N. Guseva, L. G. Mamsurova, L. V. Sazonova, and G. E. Sukhanova, Inorg. Mater. (Engl. Transl.) **16**, 996 (1980).

[13] G. V. Shamrai, R. L. Magunov, I. V. Stasenko, and A. P. Zhirnova, The Dy_2O_3-TiO_2 system, Inorg. Mater. (Engl. Transl.) **25**, 233 (1989).

[14] G. E. Sukhanova, K. N. Guseva, A. V. Kolesnikov, and L. G. Shcherbakova, Phase equilibria in the TiO_2-Ho_2O_3 system, Inorg. Mater. (Engl. Transl.) **18**, 1742 (1982).

[15] R. D. Aughterson, G. R. Lumpkin, K. L. Smith, M. de los Reyes, J. Davis, M. Avdeev, M. C. Ridgway, and J. M. Cairney, The ion-irradiation tolerance of the pyrochlore to fluorite $Ho_{(x)}Yb_{(2-x)}TiO_5$ and Er_2TiO_5 compounds: A TEM comparative study using both in-situ and bulk ex-situ irradiation approaches, J. Nucl. Mater. **507**, 316 (2018).

[16] M. A. Petrova, A. S. Novikova, and R. G. Grebenshchikov, Phase equilibria in the Er_2O_3-TiO_2 system, Dokl. Chem. (Engl. Transl.) **246**, 223 (1979).

[17] G. V. Shamrai, R. L. Magunov, I. V. Stasenko, and A. P. Zhirnova, The Tm_2O_3-TiO_2 system, Russ. J. Inorg. Chem. (Engl. Transl.) **35**, 450 (1990).

[18] G. V. Shamrai, A. V. Zagorodnyuk, R. L. Magunov, and A. P. Zhirnova, Yb_2O_3-TiO_2 system, Inorg. Mater. (Engl. Transl.) **28**, 1633 (1992).

[19] M. A. Petrova, A. S. Novikova, and R. G. Grebenshchikov, Phase equilibria in the TiO_2-Ho_2O_3 system, Inorg. Mater. (Engl. Transl.) **18**, 599 (1982).

[20] N. Mizutani, Y. Tajima, and M. Kato, Phase relations in the system Y_2O_3-TiO_2, J. Am. Ceram. Soc. **59**, 168 (1976).

[21] M. A. Petrova, A. S. Novikova, D. P. Romanov, and R. G. Grebenshchikov, Solid solutions in the Ln_2TiO_5–Lu_2TiO_5 (Ln = Gd, Tb, Er) systems, Izv. Akad. Nauk SSSR, Neorg. Mater; **22**, 1225 (1986).

[22] M. A. Petrova, A. S. Novikova, and R. G. Grebenshchikov, Phase relations in the pseudobinary systems La_2TiO_5–Lu_2TiO_5 and Gd_2TiO_5–Tb_2TiO_5, Inorg. Mater. (Engl. Transl.) **39**, 509 (2003).

[23] R. D. Aughterson, G. R. Lumpkin, K. L. Smith, Z. Zhang, N. Sharma, and J. M. Cairney, The crystal structures and corresponding ion-irradiation response for the $Tb_{(x)}Yb_{(2-x)}TiO_5$ series, Ceram. Int. **44**, 511 (2018).

[24] R. D. Aughterson, G. R. Lumpkin, K. L. Smith, and J. M. Cairn, Novel complex ceramic oxides, Ln_2TiO_5 (Ln = La, Sm, Gd, Tb, Dy, Ho, Er, and Yb), for polyphase nuclear wasteforms, J. Am. Ceram. Soc. **103**, 5536 (2020).

[25] R. D. Aughterson, G. R. Lumpkin, M. de los Reyes, B. Gault, P. Baldo, E. Ryan, K. R. Whittle, K. L. Smith, and J. M. Cairney, The influence of crystal structure on ion-irradiation tolerance in the $Sm_{(x)}Yb_{(2-x)}TiO_5$ series, J. Nucl. Mater. **471**, 17 (2016).

[26] L. M. Mentel, mendeleev - a python resource for properties of chemical elements, ions and isotopes, `https://github.com/lmmentel/mendeleev` (2014).

[27] M. Zinkevich, Thermodynamics of rare earth sesquioxides, Prog. Mater. Sci. **52**, 597 (2007).

[28] G. Adachi and N. Imanaka, The binary rare earth oxides, Chem. Rev. **98**, 1479 (1998).

[29] L. R. Morss, Thermochemical properties of yttrium, lanthanum, and the lanthanide elements and ions, Chem. Rev. **76**, 827 (1976).

[30] R. J. M. Konings, The thermodynamic properties of the f-elements and their compounds. part 2. the lanthanide and actinide oxides, J. Phys. Chem. Ref. Data **43**, 013101 (2014).

[31] Y. A. Landa, Y. A. Polonskii, B. S. Glazachev, and T. V. Milovidova, The enthalpy and specific heat of yttrium oxide at 1300-2100 °K, Refractories **15**, 86 (1974).
